# ILT4 inhibition prevents TAM- and dysfunctional T cell-mediated immunosuppression and enhances the efficacy of anti-PD-L1 therapy in NSCLC with EGFR activation

**DOI:** 10.7150/thno.52435

**Published:** 2021-01-19

**Authors:** Xiaozheng Chen, Aiqin Gao, Fang Zhang, Zijiang Yang, Shuyun Wang, Yuying Fang, Juan Li, Jingnan Wang, Wenjing Shi, Linlin Wang, Yan Zheng, Yuping Sun

**Affiliations:** 1 Department of Oncology, Jinan Central Hospital, Cheeloo College of Medicine, Shandong University, Jinan, Shandong 250013, P. R. China.; 2 Cheeloo College of Medicine, Shandong University, Jinan, Shandong 250012, P. R. China.; 3 Department of Radiation Oncology, Shandong Cancer Hospital and Institute, Shandong First Medical University and Shandong Academy of Medical Sciences, Jinan, Shandong 250012, P. R. China.; 4 Research Center of Translational Medicine, Jinan Central Hospital, Cheeloo College of Medicine, Shandong University, Jinan, Shandong 250013, P. R. China.; 5 Department of Oncology, Jinan Central Hospital affiliated to Shandong First Medical University, Jinan, Shandong 250013, P. R. China.

**Keywords:** ILT4, non-small cell lung cancer, EGFR activation, tumor-associated macrophages, T cells, immunotherapy

## Abstract

**Rationale:** Immune checkpoint inhibitors (ICIs) against the PD-1/PD-L1 pathway showed limited success in non-small cell lung cancer (NSCLC) patients, especially in those with activating epidermal growth factor receptor (EGFR) mutations. Elucidation of the mechanisms underlying EGFR-mediated tumor immune escape and the development of effective immune therapeutics are urgently needed. Immunoglobulin-like transcript (ILT) 4, a crucial immunosuppressive molecule initially identified in myeloid cells, is enriched in solid tumor cells and promotes the malignant behavior of NSCLC. However, the upstream regulation of ILT4 overexpression and its function in tumor immunity of NSCLC with EGFR activation remains unclear.

**Methods:** ILT4 expression and EGFR phosphorylation in human NSCLC tissues and cell lines were analyzed using immunohistochemistry (IHC), real-time PCR, Western blotting, immunofluorescence, and flow cytometry. The molecular signaling for EGFR-regulated ILT4 expression was investigated using mRNA microarray and The Cancer Genome Atlas (TCGA) database analyses and then confirmed by Western blotting. The regulation of tumor cell proliferation and apoptosis by ILT4 was examined by CCK8 proliferation and apoptosis assays. The impact of ILT4 and PD-L1 on tumor-associated macrophage (TAM) recruitment and polarization was evaluated using Transwell migration assay, flow cytometry, enzyme linked immunosorbent assay (ELISA) and real-time PCR, while their impact on T cell survival and cytotoxicity was analyzed by CFSE proliferation assay, apoptotic assay, flow cytometry, ELISA and cytolytic assay. Tumor immunotherapy models targeting at paired Ig-like receptor B (PIR-B, an ortholog of ILT4 in mouse)/ILT4 and/or PD-L1 were established in C57BL/6 mice inoculated with stable EGFR- overexpressing Lewis lung carcinoma (LLC) cells and in humanized NSG mice inoculated with EGFR mutant, gefitinib-resistant PC9 (PC9-GR) or EGFR-overexpressing wild type H1299 cells. PIR-B and ILT4 inhibition was implemented by infection of specific knockdown lentivirus and PD-L1 was blocked using human/mouse neutralizing antibodies. The tumor growth model was established in NSG mice injected with PIR-B-downregulated LLC cells to evaluate the effect of PIR-B on tumor proliferation. The frequencies and phenotypes of macrophages and T cells in mouse spleens and blood were detected by flow cytometry while those in tumor tissues were determined by IHC and immunofluorescence.

**Results:** We found that ILT4 expression in tumor cells was positively correlated with EGFR phosphorylation in human NSCLC tissues. Using NSCLC cell lines, we demonstrated that ILT4 was upregulated by both tyrosine kinase mutation-induced and epidermal growth factor (EGF)-dependent EGFR activation and subsequent AKT/ERK1/2 phosphorylation. Overexpressed ILT4 in EGFR-activated tumor cells induced TAM recruitment and M2-like polarization, which impaired T cell function. ILT4 also directly inhibited T cell proliferation, cytotoxicity, and IFN-γ expression and secretion. In EGFR-activated cell lines *in vitro* and in wild-type EGFR-activated C57BL/6 and humanized NSG immunotherapy models *in vivo*, either ILT4 (PIR-B) or PD-L1 inhibition enhanced anti-tumor immunity and suppressed tumor progression by counteracting TAM- and dysfunctional T cell- induced immuno-suppressive TME; the combined inhibition of both molecules showed the most dramatic tumor retraction. Surprisingly, in EGFR mutant, TKI resistant humanized NSG immunotherapy model, ILT4 inhibition alone rather than in combination with a PD-L1 inhibitor suppressed tumor growth and immune evasion.

**Conclusions:** ILT4 was induced by activation of EGFR-AKT and ERK1/2 signaling in NSCLC cells. Overexpressed ILT4 suppressed tumor immunity by recruiting M2-like TAMs and impairing T cell response, while ILT4 inhibition prevented immunosuppression and tumor promotion. Furthermore, ILT4 inhibition enhanced the efficacy of PD-L1 inhibitor in EGFR wild-type but not in EGFR mutant NSCLC. Our study identified novel mechanisms for EGFR-mediated tumor immune escape, and provided promising immunotherapeutic strategies for patients with EGFR-activated NSCLC.

## Introduction

Immune checkpoint inhibitors (ICIs) targeting the PD-1/PD-L1 axis are the milestone of anti-tumor therapy in recent years 1. These agents have achieved promising results in multiple solid tumors and have been established as the standard care of front-line therapy in advanced non-small cell lung cancer (NSCLC) 2, 3. However, the efficacy of ICIs is still limited and no more than 20% in NSCLC patients with wild-type epidermal growth factor receptor (EGFR) 4^.^ Clinical trials of ICIs in EGFR-driven NSCLC have been largely disappointing thus far 5. It has been reported that activated EGFR signaling in NSCLC cells can utilize multiple strategies to create an immunosuppressive tumor microenvironment (TME). These include impeding T cell infiltration and cytotoxicity, recruitment of suppressive tumor-associated macrophages (TAMs) and regulatory T cells (Tregs), and secretion of inhibitory cytokines and metabolites, which represent major hurdles to effective anti-tumor immunity and immunotherapy 6. Therefore, exploration of novel mechanisms for EGFR-mediated immune escape and tumor promotion and reversal of the suppressive TME is essential to improve the efficacy of ICIs in NSCLC patients with EGFR activation.

Immunoglobulin-like transcript (ILT) 4 is an inhibitory receptor of the immunoglobulin superfamily. It is mainly expressed in myeloid cells, including dendritic cells (DCs), granulocytes, monocytes, macrophages and platelets 7, 8, ILT4 in these cells represents their immunosuppressive phenotypes and negatively regulates antigen presentation of DCs, phagocytosis of neutrophils, maturation of macrophages, and platelet aggregation 9, 10. In recent years, we and others have shown that ILT4 is enriched in many solid tumor cells and directly induces their proliferation, invasion, and migration 10-14. In addition, ILT4 directs immunosuppressive T cell subset infiltration in lung adenocarcinoma patients and predicts poor patient outcome 15. These findings identify ILT4 as a potential target for tumor treatment. However, the regulatory mechanism of ILT4 expression in NSCLC cells and its functional role in anti-tumor immunity and immunotherapy remain undetermined.

TME is an integral part of cancer fundamentally orchestrating tumorigenesis, disease progression, and treatment resistance 16. Among the complex cellular components in TME, immunocytes, including TAMs and dysfunctional T cells, play central roles in ICI resistance 17. TAMs are the most abundant immune cells in the TME 18. There is substantial evidence revealing that TAMs acquire a pro-tumoral phenotype at the time of tumor initiation, and promote tumor progression by inducing T cell dysfunction, angiogenesis, and tumor cell invasion and motility 19. Immunologically, the pro-tumoral TAMs can either directly inhibit cytotoxic T lymphocyte (CTL) responses or indirectly regulate immunosuppression by reshaping the immune microenvironment 20, ultimately impairing ICI activity.

Besides TAMs, T cells including CD4^+^ T helper cells and CD8^+^ CTLs have a crucial role in tumor rejection 16. While CD4^+^ T cells kill tumor cells and recruit tumor-specific CTLs by producing IFN-γ and IL-2 21, CTLs mediate tumoricidal activity directly through the release of cytotoxic granules (perforin and granzyme) or indirectly through secretion of cytokines (IFN-γ and tumor necrosis factors) 22. However, T cells in the TME are usually hyporesponsive to tumors due to their exhausted or senescent status 23, hindering effective anti-tumor immunotherapy. Although ICIs, targeting PD-1/PD-L1 signaling, partially reverse the exhaustion of T cells 23, the mechanisms for T cell dysfunction are far more complex than previously expected. Further exploring tumor-orchestrated immunosuppression and rescuing the suppressive TME by combination immunotherapy would provide potent approaches to improve ICI efficacy. In this context, whether TAMs and dysfunctional T cells participate in ILT4-mediated tumor promotion is still unclear.

We addressed the regulator and functional role of ILT4 in NSCLC with EGFR activation and found that ILT4 was upregulated by EGFR-AKT/-ERK1/2 signaling. ILT4 induced recruitment and M2-like polarization of TAMs in NSCLC and blocked T cell infiltration and cytotoxicity. ILT4 inhibition prevented the immunosuppressive TME and tumor growth of EGFR-activated NSCLC both *in vitro* and *in vivo*. Furthermore, ILT4 blockade displayed synergy with the PD-L1 inhibitor in EGFR wild-type rather than EGFR mutant NSCLC in humanized mouse immunotherapy models. Thus, our study identified novel mechanisms for ILT4-mediated tumor immune escape in EGFR-activated NSCLC and suggested promising immunotherapeutic and combination strategies for these patients.

## Materials and Methods

### Patient tumor samples

80 tumor specimens were collected from NSCLC patients who underwent surgical resections at Jinan Central Hospital Affiliated to Shandong University from 2013.01 to 2019.12. No preoperative treatment, including chemotherapy and radiotherapy, was administered. Of the 80 samples, 28 were squamous cell carcinoma, and 52 were adenocarcinoma in which 38 harbored EGFR sensitizing mutations and 14 were EGFR wild-type. The patients were classified according to the UICC/AJCC staging system for NSCLC (8^th^ edition). The study was approved by the Institutional Ethics Committee of Jinan Central Hospital, and written informed consent was obtained from each patient.

### Tumor cell lines

NSCLC cell lines (PC9, HCC827, A549, H1299, and H1975) and immortalized human bronchial epithelial cell line (BEAS-2B) were purchased from the Cell Resource Center of the Chinese Academy of Sciences (Beijing, China). EGFR genotypes of NSCLC cell lines were as follows: PC9-exon 19 deletion, HCC827- exon 19 deletion, H1975-L858R and T790M mutations, A549 and H1299-EGFR wild-type. All cell lines were cultured in RPMI-1640 supplemented with 10% fetal bovine serum (FBS) and 1% Penicillin-Streptomycin solution. Gefitinib-resistant PC9 (PC9-GR) cells were generated by long-time exposure of PC9 cells to gradient gefitinib (Sigma-Aldrich; Cat No. SML1657). Gefitinib concentrations were increased stepwise over a dose range up to 2 μM when the growth kinetics of tumor cells resumed to a level similar to the untreated parental cells. Resistant cells were obtained approximately six months after initiation of drug exposure and eventually cultured in RPMI-1640 containing 2 μM gefitinib and 10% FBS. Its resistance to gefitinib was confirmed using the CCK8 assay according to the manufacturer's instructions (MCE; Cat No. HY-K0301).

### Preparation of conditioned medium (CM)

Tumor cells were first transfected with ILT4 knockdown lentivirus for 48h h or pretreated with control IgG (R&D system; Cat No. MAB003), ILT4 neutralizing antibody (anti-ILT4; R&D system; Cat No. MAB2078), PD-L1 inhibitory antibody (anti-PD-L1; Selleck; Cat No. A2004), or both antibodies for 24 h. The medium was replaced using serum-free medium and cells were cultured for additional 24 h. Subsequently, the supernatant was centrifuged at 1000×g for 15 min and harvested to induce TAM polarization and migration.

### Generation of human TAMs and T cells

Human peripheral blood mononuclear cells (PBMCs) were isolated by Ficoll-Paque density centrifugation from fresh whole blood donated by healthy volunteers. Human monocytes were purified from PBMCs by EasySep Human CD14 Positive Selection Kit (StemCell Technologies; Cat No.17858) and human naïve CD3^+^ T cells were purified by EasySep T cell enrichment kits (StemCell Technologies; Cat No. 19751). The purity of monocytes and naïve T cells were > 97%, as confirmed by flow cytometry. CD14^+^ monocytes were maintained in RPMI-1640 supplemented with 10% FBS and 100 ng/mL recombinant human macrophage colony stimulating factor (M-CSF) (Biolegend; Cat No. 574802) for 7 days to generate macrophages. For induction of TAMs, macrophages were seeded in 12-well plates and stimulated with 1ml mixture of CM and FBS-containing medium (1:1) for 24 h. Human naïve T cells were first activated using anti-human CD3 (Biolegend; Cat No.317326) pre-coated 6-well plates and then cultured with RPMI-1640 containing 10% FBS, 1 μg/mL anti-human CD28 (Biolegend; Cat No.302914) and 100 IU/mL recombinant human IL-2 (Pepro Tech; Cat No.2000250).

### Immunohistochemical (IHC) staining and quantification

Human and mouse lung cancer tissues were first embedded in paraffin and then sectioned into 4 μm slices. The sections were deparaffinized and rehydrated in a descending ethanol series. Following antigen retrieval, the sections were incubated with 3% hydrogen peroxide for 20 min. Tissue slides were then incubated overnight at 4 °C with following primary antibodies: anti-ILT4 antibody (1:50; Affinity; Cat No. DF9604), anti-pEGFR antibody (1:200; Abcam; Cat No. ab40815), anti-PD-L1 antibody (1:100; CST; Cat No.13684), anti-CD68 antibody (1:100; Abcam; Cat No. ab125212), anti-CD206 antibody (1:100; Abcam; Cat No. ab64693), anti-CD163 antibody (1:100; Abcam; Cat No. ab182422), anti-CD3 antibody (1:200; Abcam; Cat No. ab5690; 1:100; CST; Cat No. 85061), anti-IFN-γ antibody (1:50; Affinity; Cat No. DF6045), and anti-F4/80 antibody (1:200; Abcam; Cat No. ab111101), anti-CD80 antibody (1:500; Boster; Cat No. BM4121), anti-CD86 antibody (1:500; Proteintech; Cat No. 66406-1-Ig), anti-PIRB antibody (1:100; Immunoway; Cat No. YN1914). The two-step immunohistochemical staining kit (zsbio; Cat No. PV-9000) was used for protein expression analysis, according to the manufacturer's instructions. Finally, the slides were visualized with the 3, 3′-diaminobenzidine solution (DAB) and counterstained with hematoxylin. At least five fields were reviewed for each slide at ×400 magnification by two independent investigators in a randomized, double-blind manner. The immunoreactivity was semi-quantitatively scored according to the following scale: 0, < 5% immunoreactive cells; 1, 5-25% immunoreactive cells; 2, 25-50% immunoreactive cells; 3, 50-75% immunoreactive cells; and 4, > 75% immunoreactive cells. Staining intensity was also semi-quantitatively scored as 0 (negative), 1 (weak), 2 (intermediate), or 3 (strong). The final score for each patient was expressed as the product of the proportion and intensity scores. The cutoff scores for high and low expression were ≥ median or < median, while that for positive and negative expression were ≥ 4 or < 4. The numbers of CD3-, F4/80-, and CD206-positive cells in each specimen were also counted at ×400 magnification.

### Lentivirus, plasmid and siRNA transfection of NSCLC cells

ILT4/paired Ig-like receptor B (PIR-B)/EGFR overexpression or knockdown lentiviruses were purchased from Genechem Inc. Before infection, NSCLC cells were seeded in 6-well plates overnight, and then 1 ml fresh medium containing lentivirus (MOI: 5-10) was added to each well. After 72 h, the infection efficiency was evaluated using a fluorescence microscope and the successfully transfected cells were then screened with 2 μg/mL puromycin. The small interfering RNAs (siRNAs) targeting EGFR were purchased from GenePharma. Transfection of siRNAs in NSCLC cells was done using Lipofectamine RNAiMAX Reagents (Invitrogen; Cat No.13778030) following the manufacturer's procedures. EGFR overexpression plasmid was purchased from Genechem Inc. and transfected into tumor cells using X-treme GENE HP Reagents (Roche; Cat No. 06366546001) according to the manufacturer's instructions. Tumor cells were harvested 48-72 h after transfection for subsequent assays.

### Stimulant and inhibitor treatment of NSCLC cells

Recombinant human epidermal growth factor (EGF, Novoprotein; Cat No. DC029) or EGFR tyrosine kinase inhibitors, gefitinib (Sigma; Cat No. SML1657) and osimertinib (MCE; Cat No. HY-15772), were used to treat NSCLC cells for 24 h to activate or suppress EGFR phosphorylation. For pathway studies, ERK1/2 inhibitor U0126 (MCE; Cat No. HY-12031A), AKT1/2/3 inhibitor MK2206 (MCE; Cat No. HY-108232), and NF-κB inhibitor PDTC (MCE; Cat No. HY-18738) were applied to treat NSCLC cells for 72 h. When needed, the tumor cells were pretreated with 500 ng/mL control IgG/anti-ILT4/anti-PD-L1/ or both antibodies for 8 h, and then co-cultured with T cells for 48 h for following experiments.

### RNA extraction and real-time quantitative PCR

The tumor cells and TAMs were harvested and RNA was extracted using the total RNA extraction kit (Fastagen; Cat No. RNAfast200). The cDNA was synthesized from 2 µg purified total RNA using the HiScript III RT SuperMix for quantitative PCR (Vazyme; Cat No. R323-01) according to the manufacturer's instructions. The mRNA expression of EGFR and ILT4 in tumor cells and phenotypic markers of TAMs were determined using specific primers and analyzed using the comparative Ct method and normalized to GAPDH level. All experiments were performed in triplicate. The specific primers used are listed in Table S1.

### Western blot analysis

The NSCLC cells after transfection or drug treatment were harvested and lysed using RIPA buffer with protease inhibitor and phosphatase inhibitor cocktail. After determining the protein concentrations of cell lysates by the BCA protein assay kit, 20 μg of protein samples were separated on 10% SDS-polyacrylamide gels and transferred to PVDF membranes. The membranes were then blocked in 5% skim milk solution for 1 h and incubated with corresponding primary antibodies at 4 °C overnight. The used primary antibodies were as follows: anti-ILT4 (1:1000; Abnova; Cat No. H00010288-B01), anti-EGFR (1:1000; Abcam; Cat No.ab52894), anti-phospho-EGFR (Tyr1068) (1:1000; Abcam; Cat No. ab40815), anti-PD-L1 (1:1000; Proteintech; Cat No.66248-1-Ig), anti-PIR-B antibody (1:1000; Immunoway; Cat No. YN1914), anti-Ki67 antibody (1:1000; Abcam; Cat No. ab16667), anti-ERK1/2 (1:1000; CST; Cat No. 4695), anti-phospho-ERK1⁄2 (Thr202⁄Tyr204) (1:1000; CST; Cat No. 4370), anti-AKT (1:1000; Abcam; Cat No. ab8805), anti-phospho-AKT (Ser473) (1:1000; Abcam; Cat No. ab81283), anti-phospho-P65 (S536) (1:1000; Abcam; Cat No. ab76302) and anti-P65 (1:1000; Abcam; Cat No. ab16502). Subsequently, the membranes were washed and incubated with horseradish peroxidase-conjugated goat anti-rabbit/mouse secondary antibodies. Bands were visualized using the ECL chemiluminescent detection reagent.

### Immunofluorescence

Tumor cell lines upon EGFR overexpression/knockdown or EGF/TKI treatment were seeded on a chamber slide and fixed with 4% paraformaldehyde for 15 min. Transplanted tumor tissues from C57BL/6 mice were embedded in paraffin and sectioned into 4 μm slides for Immunofluorescence staining. The slides were deparaffinized and rehydrated in a descending ethanol series. Following antigen retrieval, the sections were incubated with 3% hydrogen peroxide for 20 min. After 1 h of blocking in donkey serum, the cells/tissue slides were incubated with primary antibodies against ILT4 (1:200; R&D; Cat No. MAB2078) and phospho-EGFR(Tyr1068) (1:200; Boster; Cat No. BM4676) or anti-F4/80 antibody (1:200; Abcam; Cat No. ab111101) and anti-CD163 antibody (1:100; Abcam; Cat No. ab182422) /anti-CD80 antibody (1:100; Boster; Cat No. BM4121) at 4 °C overnight, followed by incubation with DyLight 488 or 594 conjugated secondary antibody (1:200; Abbkine; Cat No. A23230, A23240, A23430 and A23410) for 20 min at room temperature. The cells were then counterstained with DAPI (Abbkine; Cat No. BMD00063) for 5 min. Finally, slides were mounted with an antifading medium and photographed with fluorescence microscopy. All experiments were triplicated.

### Flow cytometry analysis

The markers of human tumor cells, as well as those of human and mouse TAMs and T cells were determined by flow cytometry after surface or intracellular staining with specific antibodies conjugated with different fluorescence. For intracellular staining of IFN-γ, erythrocyte-excluded blood and spleen cells were first incubated with Cell Stimulation Cocktail plus protein transport inhibitors (eBioscience; Cat No. 4975-03) at 37 °C under 5% CO_2_ for 5 h. The following human or mouse antibodies were used: PE-anti-ILT4, PE-anti-CD163, PE-anti-CD80, PE-anti-CD86, PE-anti-CD206, APC/CY7-anti-CD45, Percp5.5-anti-F4/80, FITC-anti-CD3, Percp5.5-anti-CD4, APC-anti-CD8, and PE-anti-IFN-γ. All the antibodies were purchased from Biolegend. The stained cells were analyzed on a FACS Calibur flow cytometer (BD Bioscience) and data were analyzed using FlowJo10 software (Tree Star, Inc.; Ashland; OR).

### Microarray data analysis

H1975 cells were treated with 0.2 μM osimertinib for 24 h and then harvested in TRIzol reagent. Agilent RNA 6000 Nano Kit was used to test the RNA quality. The samples were amplified and labeled using the GeneChip WT PLUS Kit, and hybridized using the GeneChip Hybridization Wash and Stain Kit using GeneChip Hybridization Oven 645 and GeneChip Fluidics Station 450. The processed slides were scanned with the GeneChip Scanner 3000. The pathway enrichment analysis was performed based on the Kyoto Encyclopedia of Genes and Genomes (KEGG) database, and the differentially expressed genes in each pathway were verified by Fisher's exact test.

### Correlation analysis of ILT4/EGFR with TAMs or pathway molecular expression in the TCGA database

An online tool GEPIA (http://gepia.cancer-pku.cn/) was used to determine the correlation of EGFR expression with different pathway molecules (ERK/NF-κB1/P65/AKT1/JNK/P38), as well as the correlation of ILT4 with CD68 level in the TCGA database. The association of phosphorylated EGFR and MAPK/NF-κB1/AKT in the TCGA database was evaluated using an online tool cBioportal (http://www.cbioportal.org/). Pearson correlation coefficient was used to evaluate the relevance in lung adenocarcinoma and lung squamous cell carcinoma.

### Analysis of the correlation between ILT4 and immune cell infiltration in the TCGA database

To explore the correlation between ILT4 expression and immune cell subsets abundance, we utilized the immunedeconv R package 24 to make reliable immune infiltration estimations. All analyses were performed in lung adenocarcinoma and lung squamous cell carcinoma.

### TAM migration assay

To test the migration ability of TAMs, 2×10^5^ macrophages were resuspended in 200 μL of serum-free medium and plated in the upper chamber of a 24-well Transwell plate. 600 μL of indicated CM or recombined CCL2 (100 ng/mL, Novoprotein; Cat No. CM78) or CCL5 (200 ng/mL, Proteintech; Cat No. Ag25352) was added into the lower chamber as a chemoattractant. After 24 h of incubation, the cells in the upper chamber were fixed with 4% paraformaldehyde for 15 min and then stained with 0.1% crystal violet for 20 min. The non-migrated cells in the upper chamber were removed with a cotton swab. Migratory cells were counted and averaged in 3 fields at ×200 magnification with a microscope.

### Proliferation assay

For the T cell proliferation assay, anti-CD3-preactivated T cells were first labeled with CFSE (Invitrogen; Cat No. C34554) in a dilution of 1:1000. These T cells were then co-cultured with tumor cells transfected with ILT4 knockdown lentivirus or pretreated with 0.5 μg/mL IgG/anti-ILT4/anti-PD-L1/both antibodies. In some experiments, CFSE-labeled T cells were co-cultured with TAMs induced by ILT4-downregulated or control tumor cells. After 4 days of co-culture, T cells were harvested and CFSE density was measured by flow cytometry. For tumor cell proliferation, CCK8 (MCE; Cat No. HY-K0301) assay was used. Tumor cells were plated in 96-well plates at an initial number of 2×10^3^ cells/well. The absorbance of each sample was measured at 490 nm and 600 nm for 8 days. The ratio of optical density (OD) value for each group was normalized to that on Day 1 at the indicated time points. Each experiment was performed in triplicate.

### Apoptosis assay

Apoptotic cells were detected by the apoptosis assay using flow cytometry. For T cell apoptosis, anti-CD3-pre-activated T cells were co-cultured with tumor cells transfected with ILT4 knockdown lentivirus or pretreated with 0.5 μg/mL control IgG/anti-ILT4/anti-PD-L1/both antibodies for 8 h at a ratio of 2:1. The T cells were then purified with PE-anti-CD3 and apoptotic cells were detected by the Annexin V-APC/7-AAD Apoptosis Detection kit (BioLegend; Cat No. 640930). For tumor cell apoptosis, cell lines transfected with ILT4 knockdown lentiviruses were seeded into 6-well plates and cultured for 48 h. Then cells were harvested and the apoptotic rate was detected. The Annexin V-APC-positive cells (either 7-AAD-negative or -positive) were defined as apoptotic cells.

### Enzyme-linked immunosorbent assay (ELISA)

The levels of IFN-γ secreted by T cells and CCL2/CCL5 secreted by tumor cells were detected by an ELISA Kit (Bio-city; Cat No. 1110002; Boster; Cat No. EK0441, EK0494). Briefly, cell-free supernatants from tumor-T cell co-culture system or tumor CM were collected, and 100 μl/well of standards and samples were loaded into 96-well plates. After incubation with biotinylated antibodies, streptavidin-conjugated horseradish peroxidase (HRP) was added to each well and reacted with HRP substrate solution. The OD450 values were detected using Infinite M200 microplate reader (TECAN; Männedorf; Switzerland).

### T cell cytolytic assay

NSCLC cells were first pretreated with IgG, anti-ILT4 or/and anti-PD-L1 antibodies for 8 h. T cells were then co-cultured with tumor cells at different effector: target cell ratios (E/T ratios) in 12-well plates for 24 h. The cytolytic activity of T cells was quantified through the measurement of lactate dehydrogenase (LDH) concentrations and the absorbance values in culture supernatants. The percentage of specific cytotoxicity was calculated using the following formula: Cytotoxicity = (Experimental LDH release-Spontaneous LDH release)/ (Maximal LDH release-Spontaneous LDH release) ×100%. The experimental LDH release was the LDH released on co-culture of effector and target cells, whereas the spontaneous release was the LDH released from tumor cells in the absence of effector cells. The maximal LDH release represents the release after adding Triton X-100 (100% LDH release) to cells.

### *In vivo* studies

6-8-week-old female C57BL/6 and NOD-SCID IL2Rγ-null (NSG) mice were purchased from Beijing Viewsolid Bio technology Company and housed under specific pathogen-free conditions. All animal studies were approved by the animal care committee of Cheeloo College of Medicine and complied with current Chinese regulations and standards for laboratory animal use.

C57BL/6 mice were employed to evaluate PIR-B and PD-L1 blockade effects on tumor growth and immune microenvironment. Mouse Lewis lung carcinoma cells (LLC) were first transfected with EGFR overexpression lentivirus to activate EGFR, and then transfected with PIR-B knockdown or control lentivirus. 2×10^5^ differently treated LLC cells were subcutaneously injected into the right flank of C57BL/6 mice (n = 6). Anti-mouse PD-L1 (Selleck; Cat No. A2004; 200 μg/mouse) or control IgG (BioXcell; Cat No. BE0083; 200 μg/mouse) was injected intraperitoneally into each mouse every 4 days from the 7th day after tumor inoculation. Tumor volumes were measured every 4 days using a digital caliper and calculated as 0.5×length×width^2^.

To illustrate the effect of PIR-B knockdown on tumor biology, NSG mice were administered and EGFR-LLC cells were implanted into the left flank of the mice (n = 7). Tumor volumes were measured every 4 days using a digital caliper and calculated as 0.5×length×width^2^. NSG mice were used to determine the efficacy of combined ILT4 and PD-L1 blockade in EGFR wild-type or mutant NSCLC. EGFR wild-type cell line H1299 was first transfected with EGFR overexpression lentivirus to activate EGFR signaling. H1299 cells or PC9-GR cells (EGFR mutant and TKI resistant) were then transfected with lentiviruses carrying specific ILT4 or control shRNA. 3×10^6^ tumor cells were subcutaneously inoculated into the right flanks of immunodeficient NSG mice on day 0 (n = 8). On day 7, 2×10^7^ human PBMCs were separated and injected intravenously into NSG mice to establish humanized NSG mouse models. Subsequently, anti-PD-L1 (Selleck; Cat No. A2004) or control IgG (BioXcell; Cat No. BE0297) was given intraperitoneally on the same day of PBMC transplant at the dose of 200 μg/mouse. Tumor sizes were measured every 4 days.

When tumors grew to the size limit (2 cm), mice were sacrificed and tumors were isolated and weighted. The cells from the peripheral blood and spleens were isolated after lysing red blood cells for subsequent flow cytometry analysis. Tumor tissues were embedded in paraffin and dissected for IHC analysis.

### Statistical Methods

All data were expressed as mean ± SD. GraphPad Prism 8.0 was used for statistical analysis. A minimum of three independent results from cell experiments were evaluated. The correlation between EGFR and ILT4 expression in NSCLC tissues was analyzed by Spearman's correlation coefficient. The differences between two groups were verified using unpaired Student t-test, and considered statistically significant when P < 0.05 (*,* p* < 0.05; **, *p* < 0.01; ***, *p* < 0.001).

## Results

### ILT4 expression in NSCLC cells was induced by EGFR activation

**Figure 1:**
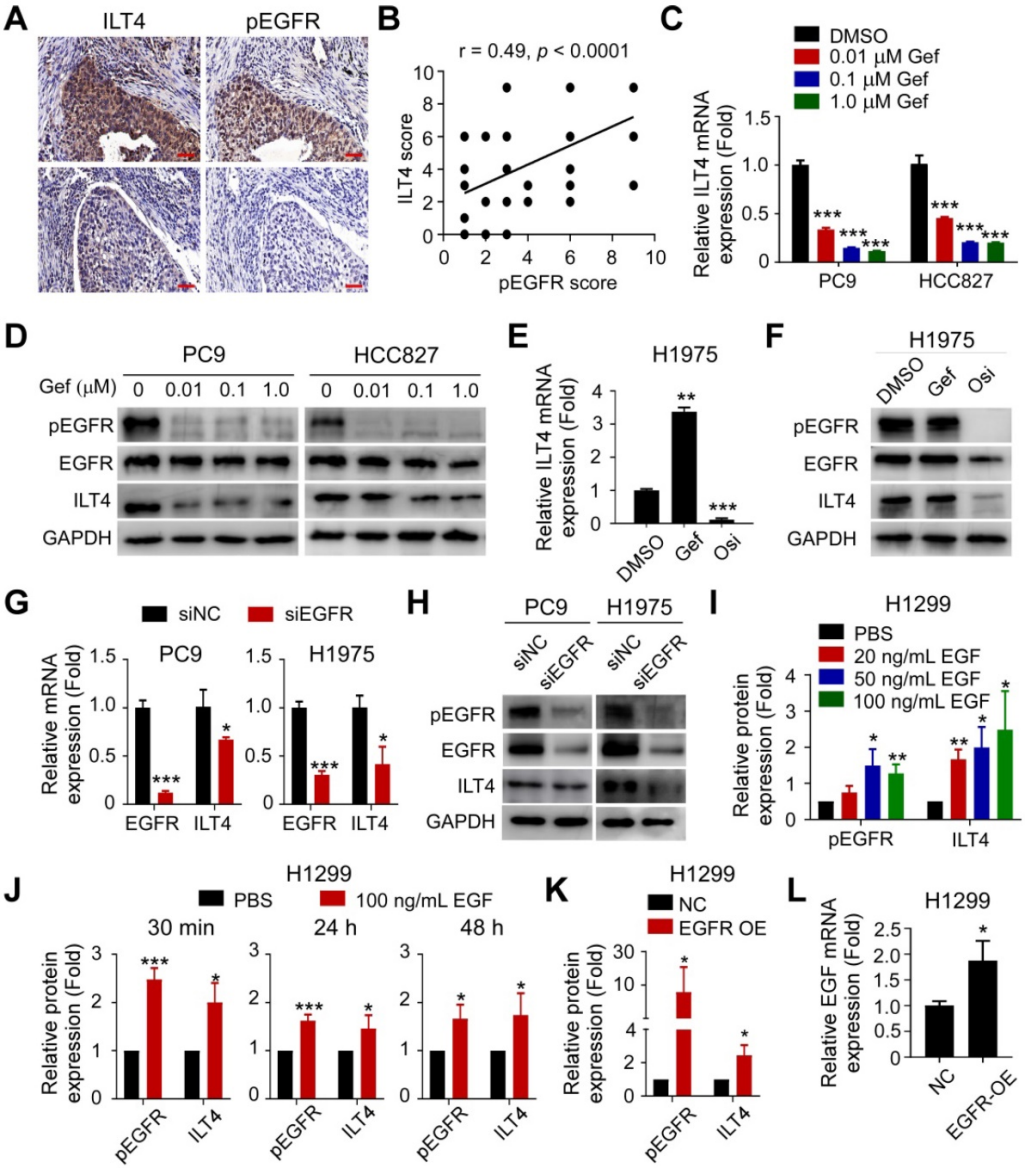
** ILT4 expression in NSCLC cells was induced by EGFR activation. (A-B)** ILT4 expression in tumor cells of NSCLC tissues was positively correlated with pEGFR levels by IHC analysis. Tumor samples were sequentially sectioned and stained with ILT4 and pEGFR primary antibodies (A) Representative images of ILT4 and pEGFR co-localization, brown granules define positive staining. (B) Statistical results from 80 patients. Scale bar: 20 µm. **(C-D)** Inhibition of EGFR activation using gefitinib significantly decreased mRNA (C) and protein (D) expression of ILT4 in a concentration-dependent manner in PC9 and HCC827 cells harboring activating EGFR mutation. PC9 and HCC827 cells were treated with a gradient concentration of gefitinib for 24 h before real-time PCR and Western blot analyses.** (E-F)** Inhibition of EGFR activation using osimertinib but not gefitinib markedly reduced mRNA (E) and protein (F) expression of ILT4 in EGFR T790M-mutant H1975 cells. The cells were treated with osimertinib (0.1 µM) or gefitinib (0.1 µM) for 24 h, and mRNA and protein expression of ILT4 were analyzed by real-time PCR and Western blotting. **(G-H)** Knockdown of EGFR in PC9 and H1975 cells decreased ILT4 mRNA (G) and protein (H) levels. PC9 and H1975 cells were transfected with specific EGFR siRNA, and the mRNA or protein expression of ILT4 and EGFR was analyzed 72 h after transfection using real-time PCR and Western blotting.** (I-J)** EGF stimulation of EGFR wild-type H1299 cells elevated ILT4 expression in both concentration- (I) and time-dependent (J) manners. (I-J) Average results from 3 independent experiments. H1299 cells were treated with different concentrations of EGF for 24 h or with 100 ng/mL EGF for different durations and Western blotting was performed to determine ILT4, EGFR, and pEGFR levels. **(K-L)** Overexpression of EGFR in H1299 cells increased pEGFR, ILT4 (L) and EGF (K) levels determined by Western blotting or real-time PCR. ILT4 and pEGFR expression were detected 48 h after transfection of EGFR overexpression plasmid using Western blotting, while EGF level was detected by real-time PCR. *, *p* < 0.05; **, *p* < 0.01; ***, *p* < 0.001. Gef: gefitinib; NC: normal control; OE: overexpression plasmid; Osi: osimertinib; si: small interfering RNA.

Our previous studies demonstrated that ILT4 is enriched in NSCLC cells 12, 15. To explore whether ILT4 was upregulated by EGFR activation, we retrospectively analyzed ILT4 expression differences in EGFR wild-type and mutant tumor tissues from 80 NSCLC patients. Surprisingly, no difference in ILT4 expression was found between these two groups (Figure S1A). Considering that EGFR can be activated in a ligand-dependent or -independent (i.e., activating EGFR mutation) manner *in vivo*, yielding phosphorylated EGFR (pEGFR), we determined the correlation of ILT4 expression with EGFR phosphorylation. We observed that in most NSCLC tissues (Figure 1A) and tumor cell lines (Figure S1B), high ILT4 expression was accompanied by increased pEGFR levels. Furthermore, ILT4 expression scores showed a positive linear correlation with pEGFR scores in human NSCLC tissues (Figure 1B). Analysis of patients' clinicopathological features with different ILT4 and pEGFR levels revealed that co-existence of ILT4 and pEGFR predicted more frequent pleural metastasis compared with double-negative patients (Table 1). These results suggested that ILT4 expression was positively correlated and might cooperate with EGFR activation to promote NSCLC progression.

To further clarify the causative connection between ILT4 expression and EGFR activation, we first downregulated ILT4 expression using specific ILT4-knockdown lentivirus and detected EGFR and pEGFR levels in PC9 and H1975 cells with intrinsic exon 19 deletion and T790M mutation, respectively. Figures S1C-D showed that ILT4 expression was markedly downregulated by specific shRNA sequences (especially by LV-shILT4-1 and LV-shILT4-3). But as shown in Figure S1E, neither EGFR nor pEGFR was affected by ILT4 downregulation. Next, we explored the regulation of ILT4 by EGFR activation. We selected PC9, HCC827, and H1975 cells that harbor activating EGFR mutations, to manipulate EGFR activation using the first-generation TKI gefitinib or the third-generation TKI osimertinib. We found that gefitinib markedly decreased mRNA and protein expression of ILT4 in PC9 and HCC827 cells in a concentration-dependent manner (Figure 1C-D). Also, osimertinib rather than gefitinib significantly downregulated ILT4 level in gefitinib-resistant but osimertinib-sensitive H1975 cells (Figure 1E-F). Using immunofluorescence and flow cytometry analyses, we further confirmed that EGFR TKIs decreased ILT4 expression in PC9 and H1975 cells (Figure S1F-G). Consistently, when we downregulated EGFR expression in PC9 and H1975 cells using specific siRNA, ILT4 expression at both mRNA and protein levels was declined (Figure 1G-H, Figure S1H-I). Given that ligand engagement is one mode of EGFR activation, we investigated the regulation of ILT4 by EGF, the classic ligand for EGFR 25, in EGFR wild-type H1299 cells. We found that ILT4 expression was upregulated by EGF stimulation in a dose-dependent manner from 0 to 100 ng/mL, with 100 ng/mL EGF causing the most significant upregulation of ILT4 expression (Figure 1I; Figure S1J-M). Furthermore, different durations of EGF stimulation also remarkably elevated ILT4 expression in H1299 cells (Figure 1J, Figure S1N). Similarly, when we upregulated the EGFR level by transfecting the EGFR overexpression plasmid in H1299 cells, EGFR phosphorylation and ILT4 expression were significantly elevated (Figure 1K, Figure S1O-R). Notably, EGF level was also augmented by EGFR overexpression (Figure 1L), suggesting that EGF binding induced by EGFR-upregulation might contribute to EGFR activation and resultant ILT4 expression. Altogether, ILT4 in NSCLC cells was induced by EGFR activation, implying a functional role of ILT4 in EGFR-activated NSCLC.

### Activated ERK and AKT signaling mediated EGFR-driven ILT4 expression

**Figure 2:**
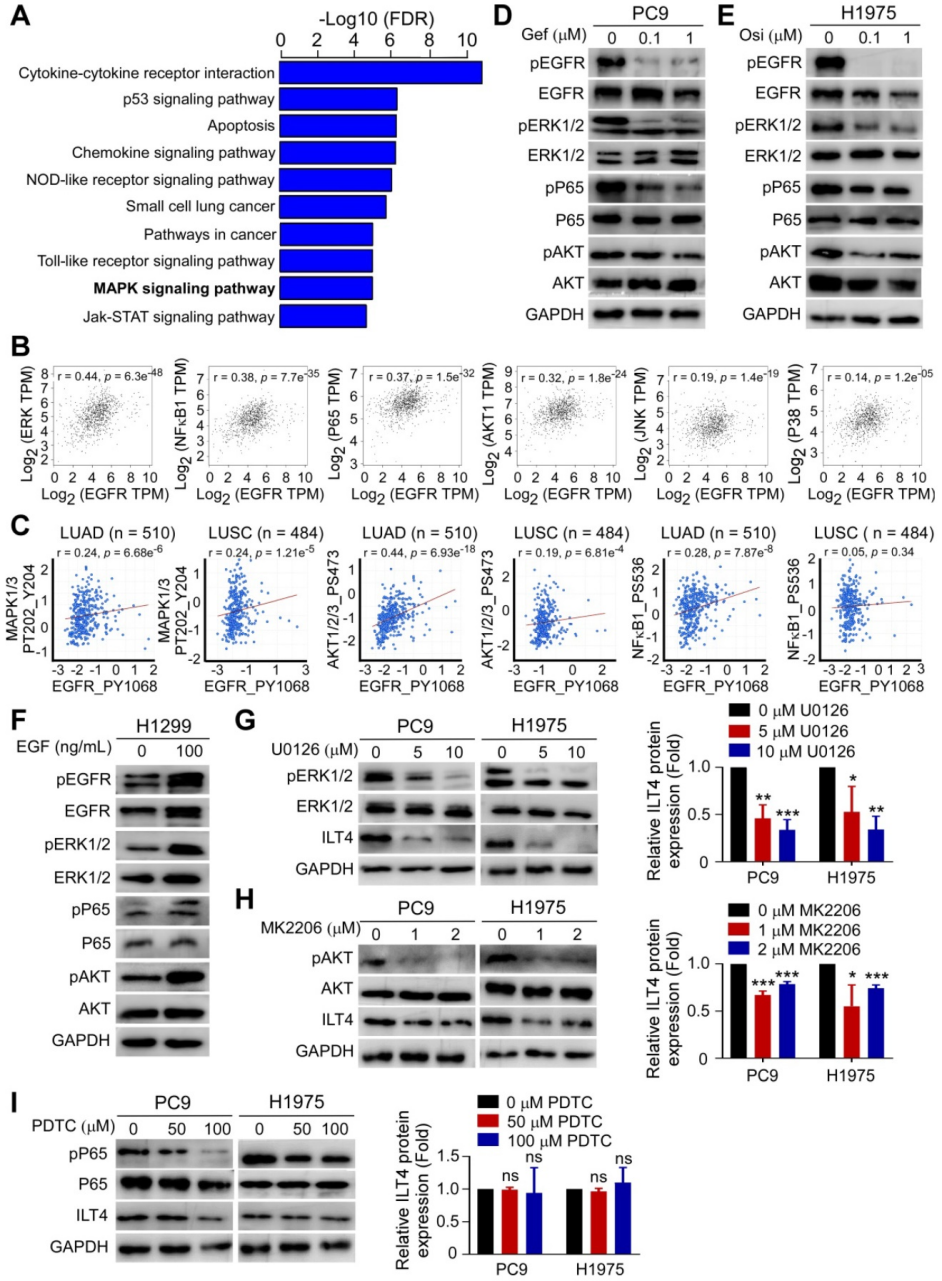
** Activated ERK and AKT signaling mediated EGFR-induced ILT4 expression. (A)** MAPK was among the most significantly altered signaling pathways upon treatment with osimertinib in H1975 cells with osimertinib (0.2 µM) for 24 h. mRNA was extracted and microarray was performed for cluster analysis of altered signaling pathways.** (B)** EGFR expression in NSCLC tissues was positively correlated with EKR (r = 0.44), NF-κB1 (r = 0.38), P65 (r = 0.37), and AKT1(r = 0.32) in the TCGA database. The online tool GEPIA was used to analyze the correlation of EGFR with key modulators of MAPK, NF-κB, and AKT signaling pathways. A correlation coefficient (r) of > 0.3 was considered significant. (**C**) pEGFR expression in lung adenocarcinoma and squamous cell carcinoma was positively correlated with activation of MAPK1/3, AKT1/2/3, and NF-κB1 in the RPPA database. The online tool cBioportal and Spearman correlation coefficient were used to evaluate the relevance. **(D-E)** Treatment with a gradient concentration of gefitinib or osimertinib inhibited ERK1/2, P65, and AKT phosphorylation in PC9 (D) or H1975 (E) cells. PC9 or H1975 cells were treated with different concentrations of gefitinib or osimertinib for 24 h and Western blotting was performed to determine the phosphorylation of signaling molecules. **(F)** EGF stimulation activated ERK1/2, P65, and AKT phosphorylation in H1299 cells treated with 100 ng/mL EGF for 24 h before Western blotting. **(G-I)** Treatment with ERK (U0126) or AKT (MK2206) inhibitor rather than NF-κB (PDTC) inhibitor decreased ILT4 expression in both PC9 and H1975 cells treated with different concentrations of specific inhibitors for 72 h and Western blotting was performed. The average results from 3 independent experiments are shown. Gef: gefitinib; Osi: osimertinib.

To probe into the underlying mechanisms of EGFR-driven ILT4 expression, we assessed the altered downstream signaling in osimertinib-treated H1975 cells by mRNA microarray analysis. As shown in Figure 2A, MAPK was among the most significantly affected signaling pathways upon EGFR inhibition in H1975 cells. MAPK, NF-κB, and AKT were reported to be the classic signaling pathways downstream of EGFR activation 26. We searched the TCGA database and determined the correlation of EGFR/phosphorylated EGFR with key modulators of MAPK, NF-κB, and AKT pathways including ERK, P38, JNK, NF-κB1, P65, and AKT. The results showed remarkable positive correlation of EGFR/phosphorylated EGFR with ERK, NF-κB1, P65, and AKT (Figure 2B-C). Using Western blotting, we confirmed TKIs' effect on the phosphorylation of ERK, P65, and AKT.As presented in Figure 2D-E, treatment with gefitinib and osimertinib inhibited the phosphorylation of ERK1/2, P65, and AKT in PC9 and H1975 cells, respectively. Consistently, EGF stimulation augmented EGFR phosphorylation in H1299 cells (Figure 2F). Next, we examined whether these signaling pathways were responsible for ILT4 upregulation. We treated PC9 and H1975 cells with specific ERK (U0126), NF-κB (PDTC) or AKT (MK2206) inhibitors, and evaluated ILT4 expression by Western blotting. The results showed that inhibition of ERK or AKT, but not NF-κB signaling, decreased ILT4 expression in PC9 and H1975 cells (Figure 2G-I). These results revealed that EGFR activation induced ILT4 expression through ERK and AKT signaling pathways in NSCLC cells.

### ILT4 in EGFR-activated tumor cells promoted TAM recruitment and M2-like polarization

Our previous study demonstrated that ILT4 promoted NSCLC malignancy and contributed to tumor progression 12. To further examine the role of ILT4 in the pathogenesis of EGFR-activated NSCLC, we examined the proliferation and apoptosis of tumor cells upon ILT4 knockdown. As shown in Figure S2A-B, ILT4 knockdown inhibited the proliferation and Ki-67 expression of PC9 and H1975 cells but induced their apoptosis (Figure S2C-D). These results indicated that ILT4-regulated biological function accelerated tumor growth of EGFR-activated NSCLC.

Our previous study also suggested that ILT4 might be a potential checkpoint molecule in tumor immunotherapy 10, but the precise regulation of immunosuppressive TME by ILT4 was unclear. Given the abundance and pivotal role of TAMs and T cells in immunosuppression 19, we questioned whether ILT4 impacted TAM- and T cell-mediated tumor immune evasion. We first searched the TCGA database to explore the correlation between ILT4 and infiltration of macrophages and T cell subsets and found that ILT4 expression was positively correlated with CD68 level (Figure S3A). Then, we confirmed the correlation of ILT4 level with M2-like macrophage infiltration in both LUAD and LUSC cohorts utilizing the immunedeconv R package (Figure S3B-C). We also found that the high ILT4 level predicted decreased CD4^+^ non-regulatory and CD8^+^ T cells (Figure S3D-F). These results indicated that ILT4 might regulate the accumulation and function of TAMs and T cells.

**Figure 3:**
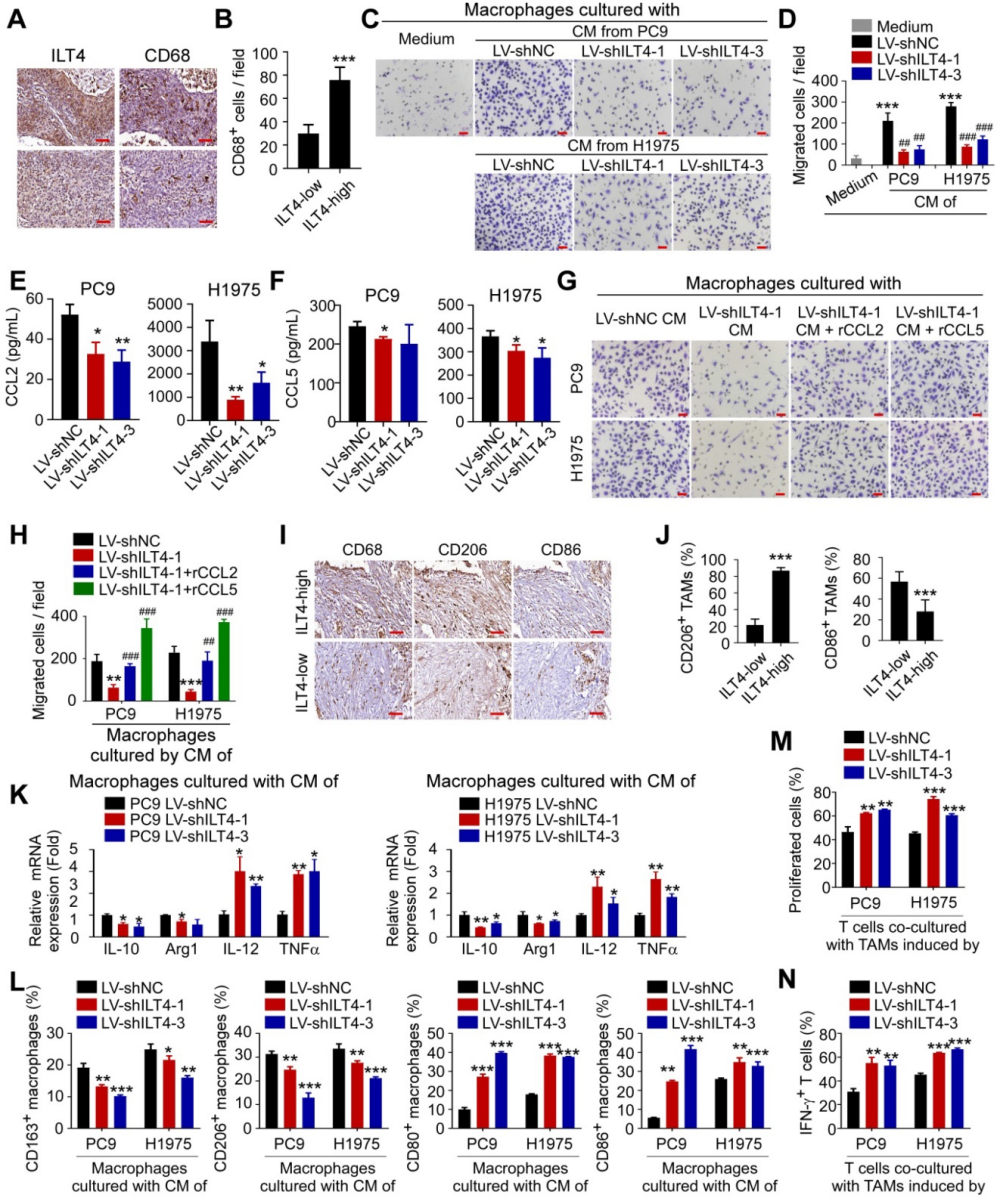
** ILT4 in EGFR-activated tumor cells promoted TAM recruitment and M2-like polarization. (A-B)** Patients with high ILT4 expression in tumor cells of NSCLC tissues showed markedly elevated infiltration of CD68^+^ TAMs by IHC analysis. (A) Representative images of ILT4 expression and TAM infiltration, brown granules represent positive staining. (B) Average results from 80 patients. ILT4-low and -high were defined by IHC score < 6 (median) and ≥ 6. Scale bar: 20 µm. **(C-D)** TAMs induced by PC9 and H1975 cells displayed increased migration ability compared with parental macrophages, whereas ILT4 knockdown in PC9 and H1975 cells restricted tumor-induced TAM migration. Tumor cells were first transfected with lentivirus carrying ILT4 shRNA for 48 h, and CM was collected for macrophage culture and TAM induction. The migration ability of TAMs was evaluated by the Transwell migration assay. (C) Images of migrated cells and (D) Average results from 3 independent experiments. Scale bar: 50 µm. *, *p* < 0.05; **, *p* < 0.01 compared with the medium group; ***, *p* < 0.001. ^###^, *p* < 0.001 compared with the LV-shNC group. **(E-F)** ILT4 knockdown in PC9 and H1975 cells decreased the secretion of CCL2 and CCL5. (E) showed ELISA results of CCL2, (F) showed CCL5. **(G-H)** Recombinant human CCL2 or CCL5 reversed ILT4 knockdown and decreased macrophage migration. 100 ng/mL recombinant human CCL2 or 200 ng/mL CCL5 were added to the CM of ILT4 knockdown PC9 and H1975 cells to induce TAM migration. The migration ability of TAMs was evaluated by the Transwell migration assay. (G) Images of migrated cells. (H) Average results from 3 independent experiments. Scale bar: 50 µm. *, p < 0.05; **, p < 0.01 compared with the medium group; ***, p < 0.001. compared with LV-shNC group. **(I-J)** Patients with high ILT4 expression in tumor cells displayed more frequent CD206^+^ but less CD86^+^ TAM accumulation in tumor tissues by IHC analysis. (I) Representative images of CD68^+^ TAMs, CD206^+^ M2-like TAMs and CD86+ M1-like TAMs frequencies; brown granules represent positive staining. (J) Average results from 80 patients. ILT4-low and -high were defined by IHC score < 6 and ≥ 6 respectively. Scale bar: 20 µm. **(K-L)** ILT4 knockdown in PC9 and H1975 cells decreased M2-like markers (CD163, CD206, IL-10 and Arg1) and increased M1-like markers (CD80, CD86, IL-12 and TNFα) in TAMs by real-time PCR (K) and flow cytometry (L). TAMs were induced by CM as in (D) for 24 h. **(M-N)** TAMs induced by ILT4-downregulated PC9 and H1975 cells promoted the proliferation (M) and IFN-γ expression (N) of T cells. For T cell proliferation assay, CD3^+^ T cells separated from fresh PBMCs were first pre-activated by anti-CD3 for 24 h and stained with CFSE (1:1000), and then co-cultured for 4 days with TAMs induced by CM of ILT4-downregulated PC9 and H1975 cells. Flow cytometry was performed to determine CFSE strength. For IFN-γ expression, pre-activated CD3^+^ T cells were co-cultured with TAMs as described above for 48 h and IFN-γ levels were analyzed by flow cytometry. *, *p* < 0.05; **, *p* < 0.01; ***, *p* < 0.001. Arg1: arginase 1; CM, conditioned medium; LV-shILT4: lentivirus carrying ILT4 shRNA; LV-shNC: lentivirus carrying control shRNA.

Next, we stained ILT4 and CD68 (TAM marker) in sequential sections of 80 NSCLC tissues and found that patients with high ILT4 expression in tumor cells displayed markedly elevated CD68^+^ TAM infiltration (Figure 3A-B). We also purified CD14^+^ monocytes from PBMCs of healthy volunteers to generate macrophages, and induced macrophages to TAMs using CM from ILT4-downregulated or control tumor cell lines. The migration ability of TAMs was evaluated by the Transwell assay. As expected, macrophages cultured with CM of both PC9 and H1975 cells showed enhanced migration ability compared with those cultured with normal medium, while ILT4 knockdown in tumor cells partially prevented TAM migration (Figure 3C-D). Since chemokines and cytokines including CCLs (chemokine (C-C motif) ligands), CXCLs (Chemokine (C-X-C motif) ligands), EGF and CSFs (Colony stimulating factors) are important drivers for macrophage recruitment 27, 28, we examined their expression in tumor cells upon ILT4 knockdown. The expression of classic macrophage chemokines including CCL8, CXCL1, CXCL9-11, EGF, CSF1 was not consistently altered upon ILT4 downregulation in PC9 and H1975 cells. But ILT4 knockdown significantly decreased the expression and secretion of CCL2 and CCL5 in both PC9 and H1975 cells (Figure S3G, Figure 3E-F). However, treatment with recombinant human CCL2 (rCCL2) or CCL5 (rCCL5) reversed TAM migration decreased by ILT4-downregulated tumor cells (Figure 3G-H). These results indicated that ILT4 promoted CCL2 and CCL5 secretion, which are responsible for the migration and recruitment of TAMs into the TME. Upon recruitment into the TME, macrophages shift their functional phenotypes ranging from classical M1 to alternative M2 macrophages in response to various microenvironmental signals from tumor cells. The M1 macrophages, with high levels of CD80, CD86, IL-12, and TNFα, were involved in the inflammatory response, pathogen clearance, and antitumor immunity. In contrast, the M2 macrophages, expressing high levels of CD163, CD206, IL-10, and Arginase 1, showed an anti-inflammatory response, wound healing, and pro-tumorigenic properties. TAMs, closely resembling the M2-polarized macrophages, were critical modulators of the tumor microenvironment 29. We investigated ILT4-regulated TAM polarization and found a markedly increased frequency of CD206^+^ (M2-like TAM marker) but decreased CD86^+^ (M1-like TAM marker) TAMs in patients with high ILT4 expression compared to those with low ILT4 levels (Figure 3I-J). We then examined the changes in M2-like TAM markers (CD163, CD206, CD209, IL-10 and Arginase 1) and M1-like TAM markers (CD80, CD86, IL-12, and TNFα) induced by CM from ILT4-downregulated tumor cells. We observed that ILT4 knockdown in tumor cells decreased M2-like markers, including CD163, CD206, IL-10, and Arginase 1 but increased M1-like markers including CD80, CD86, IL-12, and TNFαin TAMs (Figure 3K-L, Figure S3H). These data suggested that ILT4 in tumor cells induced M2-like polarization of TAMs. To further determine whether ILT4 modulated TAM-induced T cell immunity, we co-cultured T cells with TAMs induced by ILT4-downregulated PC9 and H1975 cells and determined T cell proliferation ability and IFN-γ expression level by flow cytometry. The ILT4 knockdown in both tumor cell lines promoted TAM-mediated T cell proliferation (Figure 3M, Figure S3I) and IFN-γ generation (Figure 3N, Figure S3J). Thus, ILT4 in EGFR-activated tumor cells promoted the accumulation and M2-like polarization of TAMs and facilitated TAM-induced T cell dysfunction.

### ILT4 in EGFR-activated tumor cells directly impaired the proliferation and cytotoxicity of T cells

**Figure 4:**
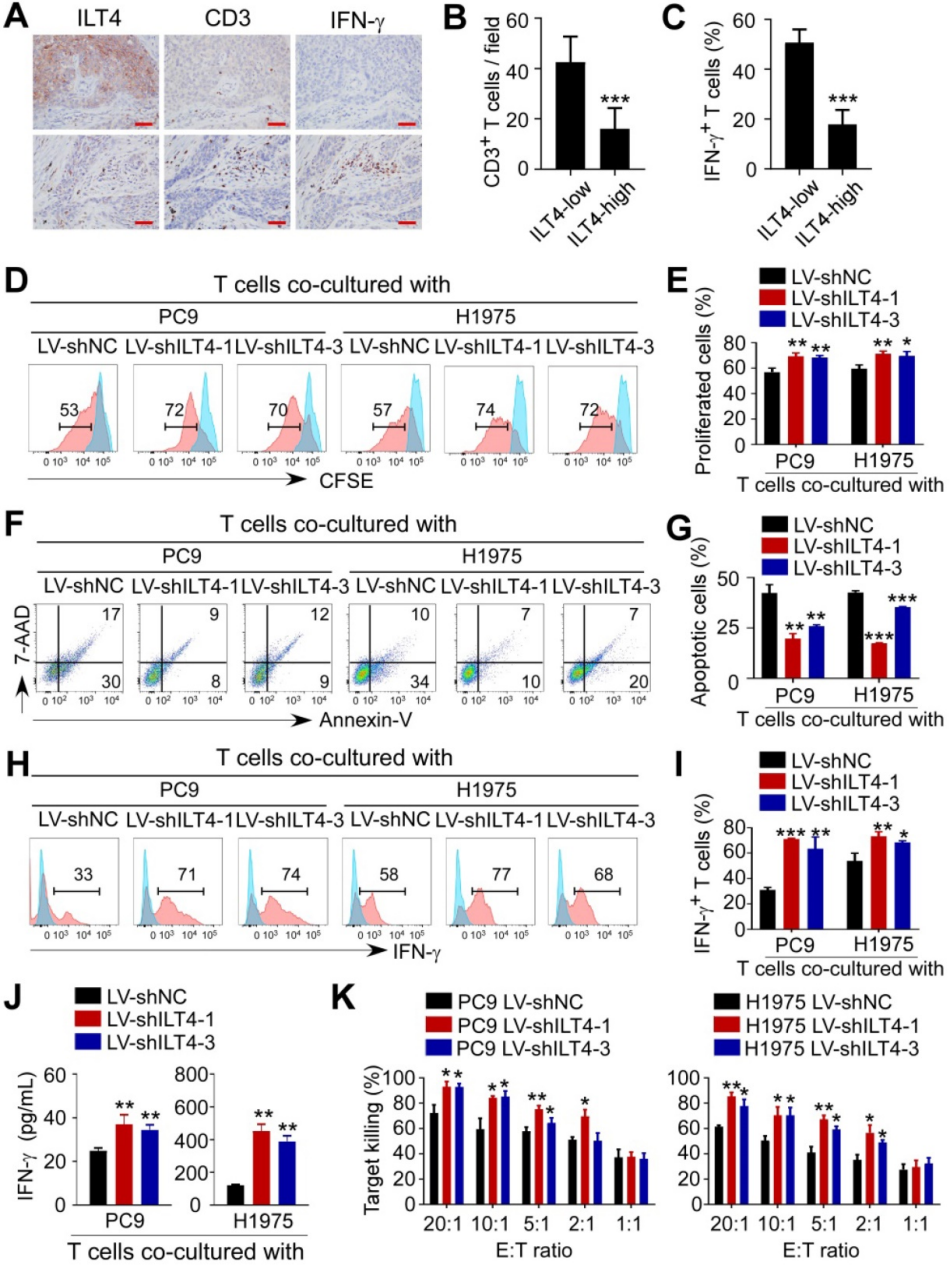
** ILT4 in EGFR-activated tumor cells impaired the proliferation and cytotoxicity of T cells. (A-B)** High ILT4 expression in tumor cells of NSCLC tissues was correlated with decreased infiltration and IFN-γ generation in CD3^+^ T cells detected by IHC analysis. (A) Images of ILT4 expression, T cell infiltrates, and IFN-γ levels, brown granules represent positive staining. (B) Average numbers of T cells in 80 patients. The cutoff scores for ILT4-high and -low were the same as in Figure 3I. Scale bar: 20 µm. **(C)** Patients with high ILT4 expression displayed significantly decreased IFN-γ levels in tumor-infiltrating T cells compared with ILT4-low patients by IHC analysis. The histogram shows the average proportion of IFN-γ^+^ T cells from 80 patients.** (D-E)** T cells co-cultured with ILT4-downregulated PC9 and H1975 cells showed increased proliferation ability compared with the counterpart group. PC9 and H1975 cells were first transfected with ILT4-knockdown lentivirus for 48 h, and then co-cultured with CD3^+^ T cells for 4 days at 1:2 ratio. The proliferation ability of T cells was assessed by the CFSE proliferation assay. (D) Flow cytometry results and (E) Average results from 3 independent experiments. **(F-G)** Transfection of ILT4-knockdown lentivirus in PC9 and H1975 cells inhibited T cell apoptosis compared with the control lentivirus group. Flow cytometry was performed to assess apoptotic T cells after co-culturing with different tumor cells. (F) Representative results for T cell apoptosis and (G) Results from 3 independent experiments. **(H-I)** ILT4 knockdown in PC9 and H1975 cells increased IFN-γ production in T cells. T cells co-cultured with tumor cells were collected and evaluated by flow cytometry to determine IFN-γ expression levels. (H) Flow cytometry results and (I) Average results from 3 independent experiments.** (J)** T cells co-cultured with ILT4-downregulated PC9 and H1975 cells released more IFN-γ into the supernatant than those co-cultured with control tumor cells. IFN-γ secretion in the same supernatant as in (F-G) by ELISA. **(K)** T cells co-cultured with ILT4-downregulated PC9 and H1975 cells displayed increased cytolytic ability compared with those co-cultured with control tumor cells. T cells were co-cultured with ILT4-downregulated or control PC9 and H1975 cells at different E: T ratios for 4 days, and the cytolysis assay was used to assess the killing ability of T cells. *, *p* < 0.05; **, *p* < 0.01; ***, *p* < 0.001. E:T ratio: Effector: target cell ratio; LV-shILT4: lentivirus carrying ILT4 shRNA; LV-shNC: lentivirus carrying control shRNA.

Given the significant correlation of ILT4 expression with T cell infiltration in the TCGA database, we explored the direct regulation of ILT4 on T cell immunity. We first confirmed the correlation between ILT4 level and T cell infiltration and IFN-γ expression in our patient cohort by co-staining ILT4, CD3, and IFN-γ in sequential tissue sections. As shown in Figure 4A-C, ILT4 expression in tumor cells was inversely correlated with CD3^+^ T cell density and IFN-γ production. Next, we established a tumor-T cell co-culture system to assess the role of tumor cell ILT4 on T cell survival and cytotoxicity. We discovered that when T cells were co-cultured with ILT4-downregulated PC9 and H1975 cells, their proliferation ability was significantly increased (Figure 4D-E) but apoptosis was markedly decreased (Figure 4F-G). Besides increased cell numbers, IFN-γ expression (Figure 4H-I) and secretion (Figure 4J) by T cells were also elevated upon ILT4 downregulation in PC9 and H1975 cells, suggesting that T cell functions were perturbed by ILT4 overexpression. We also used cytolytic assay to verify the ILT4-regulated T cell killing ability. PC9 and H1975 cells with/without ILT4-downregulation were co-cultured with pre-activated T cells in different effector to target ratios, and after 24 h, LDH concentration was detected in the supernatants. The results showed that ILT4 knockdown in tumor cells heightened the killing ability of T cells (Figure 4K). These results demonstrated that ILT4 in EGFR-activated tumor cells directly inhibited the survival and killing ability of T cells.

### ILT4 blockade in EGFR-activated NSCLC cells acted synergistically with PD-L1 inhibitor to reverse TAM- and dysfunctional T cell-mediated immunosuppression

We have illustrated that ILT4 in EGFR-activated tumor cells impeded T cell immunity indirectly through the accumulation of M2-like TAMs and directly by inhibiting T cell survival and cytotoxicity, which might be the main causes of ICI resistance. We also observed significant co-existence of ILT4 and PD-L1 in most NSCLC tissues (Table 1). Therefore, we explored the effect of ILT4 blockade on ICI efficacy. We first analyzed the migration and M2-like markers of TAMs induced by anti-ILT4- and/or anti-PD-L1-pretreated PC9 and H1975 cells. We found that CM from either anti-ILT4- or anti-PD-L1-pretreated tumor cells inhibited TAM migration while combined application of both antibodies showed the most dramatic inhibition (Figure S4A-B). Moreover, anti-ILT4 or anti-PD-L1 treatment markedly decreased the expression and secretion of CCL2 and CCL5 in both tumor cell lines with a synergistic effect in the combination treatment group (Figure S4C-F). Meanwhile, CM from anti-ILT4- or anti-PD-L1-pretreated tumor cells decreased CD163 and CD206 levels in TAMs, and combined antibody group displayed the lowest CD163 and CD206 expression (Figure S4G-I). These results suggested that anti-ILT4 and anti-PD-L1 had a synergistic impact on TAM recruitment and M2-like polarization.

We further evaluated ILT4 and PD-L1 blockade on T cell survival and cytotoxicity. As shown in Figure S4J-K, pretreatment of tumor cells using either anti-ILT4 or anti-PD-L1 promoted the proliferation of T cells co-cultured with these tumor cells, and combined antibodies yielded most significant improvement in T cell proliferation. On the contrary, co-culturing with anti-ILT4- or anti-PD-L1-pretreated tumor cells inhibited T cell apoptosis with the most significant effect in the combination antibody group compared with the IgG pretreatment group (Figure S4L-M). Functionally, co-culturing with anti-ILT4- or anti-PD-L1-pretreated tumor cells significantly elevated the IFN-γ levels and tumor eradication by T cells, while combined blockade of both molecules displayed the most remarkable increase (Figure S4N-O). These results suggested that ILT4 blockade might be a desirable approach to improve ICI efficacy in EGFR-activated NSCLC.

**Figure 5:**
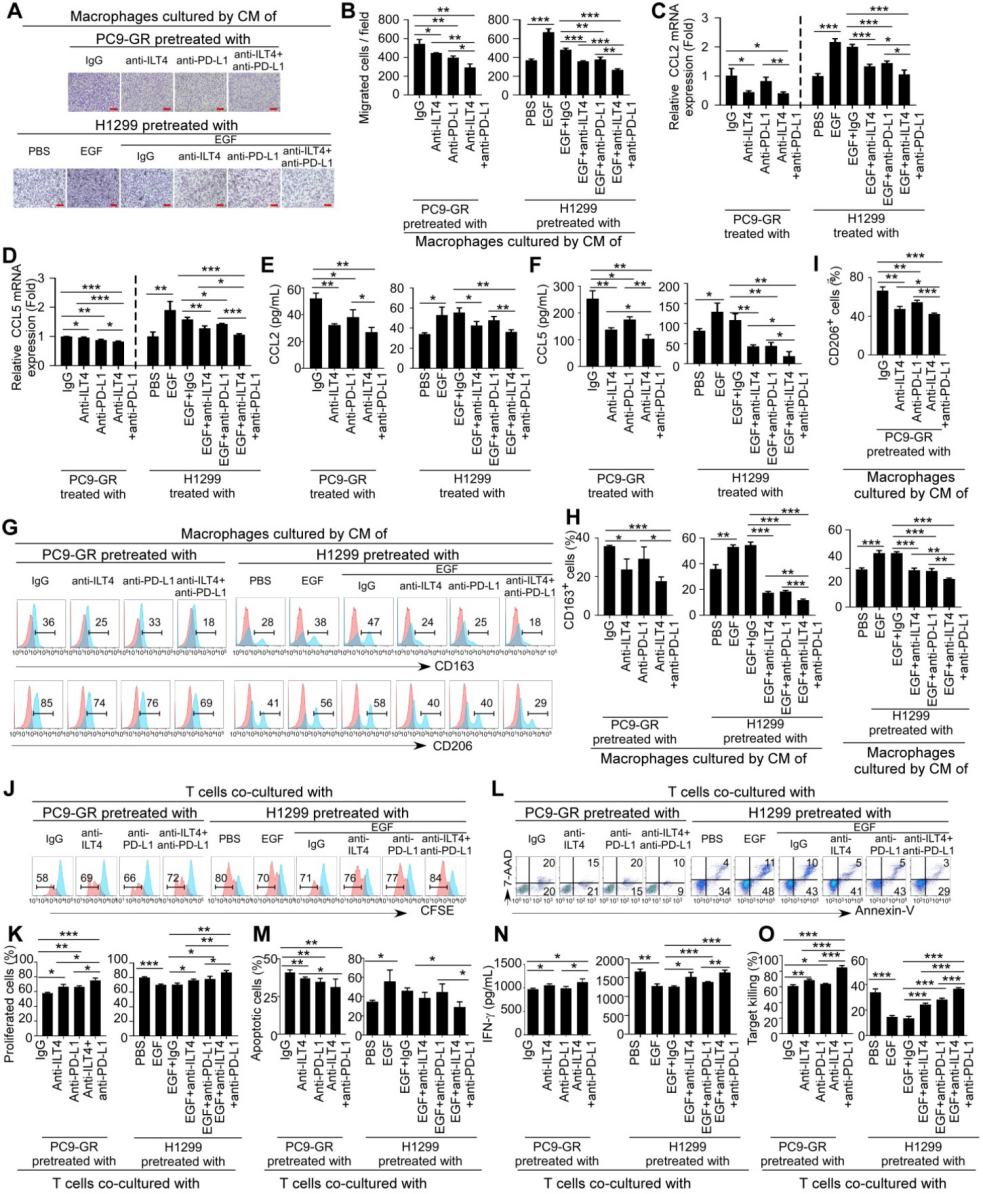
** Combined ILT4 and PD-L1 blockade in EGFR-activated NSCLC cells synergistically improved TAM- and dysfunctional T cell-mediated immunosuppression. (A-B)** ILT4 or PD-L1 blockade in PC9-GR and H1299 cells inhibited the migration ability of TAMs, while combined ILT4 or PD-L1 blockade showed a stronger suppressive effect compared with either antibody alone. PC9-GR and EGF-preactivated H1299 cells were first pretreated with anti-ILT4 (500 ng/mL)/anti-PD-L1 (500 ng/mL)/combined anti-ILT4 (250 ng/mL) and anti-PD-L1 (250 ng/mL) for 8 h. CM from these cells was collected for macrophage culture and TAM induction. The migration ability of TAMs was evaluated by the Transwell migration assay. (A) Images of TAM migration, and (B) Average results from 3 independent repetitions. Scale bar: 50 µm.** (C-F)** ILT4 or PD-L1 blockade in PC9-GR and EGF-preactivated H1299 cells decreased the expression and secretion of CCL2 and CCL5, while combined blockade showed a synergistic effect. (C-D) Decreased CCL2 and CCL5 mRNA expression detected by real-time PCR, (E-F) Decreased CCL2 and CCL5 secretion detected by ELISA. Tumor cells were treated and conditioned media were collected as described in (A).** (G-I)** ILT4 or PD-L1 blockade in PC9-GR and EGF-preactivated H1299 cells reversed tumor-induced M2-like markers in TAMs, while combined ILT4 and PD-L1 blockade displayed more significant reversion compared with either blockade alone. TAMs were induced as described in (A), and M2-like markers in TAMs were determined by flow cytometry. (G) Flow cytometry results and (H-I) Average results of CD163 and CD206 from 3 independent experiments. **(J-K)** ILT4 or PD-L1 blockade in PC9-GR and EGF-preactivated H1299 cells increased T cell proliferation, while combined ILT4 or PD-L1 blockade showed a synergistic effect on T cell proliferation. PC9-GR and H1299 cells were treated as mentioned in (A), and co-cultured with anti-CD3-preactivated CD3^+^ T cells for 4 days at the ratio of 1:2. CFSE proliferation assay was performed to determine the proliferation of T cells. (J) Images of flow cytometry and (K Average results from 3 independent experiments. **(L-M)** ILT4 or PD-L1 blockade in PC9-GR and EGF-preactivated H1299 cells suppressed T cell apoptosis, while combined ILT4 and PD-L1 blockade showed a more significant suppression than either antibody alone. T cells co-cultured with tumor cells for 2 days were evaluated for apoptotic T cells by flow cytometry. (L) Flow cytometry results and (M) Average results from 3 independent experiments. **(N)** ILT4 or PD-L1 blockade in PC9-GR and EGF-preactivated H1299 cells promoted IFN-γ secretion in co-cultured T cells, while combined blockade released more abundant IFN-γ level than either antibody alone. T cells as in (J) were evaluated for IFN-γ level by ELISA.** (O)** T cells co-cultured with PC9-GR and H1299 cells pretreated with anti-ILT4 or anti-PD-L1 had stronger tumoricidal ability compared with those co-cultured with IgG-pretreated tumor cells, and T cells co-cultured with combination antibody-pretreated tumor cells showed the strongest tumor cytolytic activity. Tumor cells were pretreated with different antibodies as mentioned in (A) and then co-cultured with tumor cells at an E:T ratio of 5:1. Cytolysis assays were employed to determine the killing ability of T cells. *, *p* < 0.05; **, *p* < 0.01; ***, *p* < 0.001. Anti: neutralization antibody; CM: conditioned medium.

Two distinct mechanisms for EGFR activation are observed in NSCLC patients. One is intrinsic activation caused by EGFR mutation, and the other is extrinsic activation by ligand engagement in EGFR wild-type patients 30. ICI treatment in both populations is clinically challenging. For the EGFR-mutant subtype, although EGFR-TKIs achieved excellent results as the front-line therapy 31, treatment options for patients who acquire resistance to EGFR-TKIs are limited and ICI treatment in these patients is ineffective 32. Moreover, the benefit of ICIs in EGFR wild-type subtype is also limited with a response rate of no more than 20% 4. Therefore, there is an urgent need to improve ICI activity in NSCLC patients. To explore the impact of ILT4 blockade on the efficacy of PD-L1 inhibitor in TKI-resistant NSCLC, we first established gefitinib-resistant PC9 (PC9-GR) cells by long-term stimulation of parental PC9 cells with low-dose gefitinib. Using the CCK8 assay, we verified the resistance of PC9-GR to regular dose of gefitinib with an IC50 of 15.16 μM in PC9-GR cells relative to 0.018 μM in parental PC9 cells (Figure S5A). Also, gefitinib treatment inhibited ILT4 expression and EGFR phosphorylation in PC9 but not in PC9-GR cells (Figure S5B-C). There were comparable levels of pEGFR in PC9 and PC9-GR cells, suggesting that PC9-GR was still EGFR-dependent (Figure S5C). Next, we assessed the impact of ILT4 blockade to ICI treatment in PC9-GR cells and EGF-preactivated H1299 (EGF-H1299) cells. TAMs were induced by culturing macrophages with CM of PC9-GR or EGF-H1299 cells that were pretreated with anti-ILT4 and/or anti-PD-L1, and their migration ability was determined by the Transwell migration assay. As shown in Figure 5A-B, pretreatment of PC9-GR with either anti-ILT4 or anti-PD-L1 dramatically inhibited TAM migration, while blockade of both molecules most significantly suppressed TAM migration. Similarly, EGF stimulation of H1299 cells remarkably increased the migration ability of TAMs, whereas the addition of either anti-ILT4 or anti-PD-L1 or combined antibodies markedly decreased TAM migration with the combination group showing the most significant inhibition (Figure 5A-B). We then examined the CCL2 and CCL5 levels in PC9-GR and EGF-H1299 cells upon anti-ILT4 or anti-PD-L1 treatment. As expected, treatment with either antibody markedly decreased the expression and secretion of CCL2 and CCL5 in these tumor cells, with the most significant decrease in the combination treatment group (Figure 5C-F). We also determined TAM phenotypes following ILT4 and PD-L1 blockade, in PC9-GR or EGF-H1299 and observed reduced M2-like markers (CD163 and CD206) in TAMs, and the reduction was highest with both antibody treatment (Figure 5G-I). These data suggested that combined ILT4 and PD-L1 blockade synergistically prevented recruitment and M2-like polarization of TAMs in both EGFR-TKI resistant and EGFR wild-type NSCLC and might cooperate to repress TAM-mediated tumor promotion.

Besides TAMs, we examined the ILT4 and PD-L1 blockade-modulated T cell immunity. PC9-GR or EGF-H1299 cells were pretreated with anti-ILT4, anti-PD-L1, or combined antibodies for 8 h. Subsequently, these tumor cells were co-cultured with anti-CD3-preactivated CD3^+^ T cells and T cell proliferation and apoptosis were detected 96 h and 48 h after co-culture respectively. Figure 5J-K shows that T cells co-cultured with anti-ILT4- or anti-PD-L1-pretreated tumor cells had higher proliferation rate than those cocultured with IgG-pretreated tumor cells, while pretreatment of tumor cells with combined antibodies led to most significant increase in T cell proliferation. In contrast, pretreatment of tumor cells with either anti-ILT4 or anti-PD-L1 antibodies yielded lower apoptosis of T cells compared with the control IgG group, and the combination treatment generated the least number of apoptotic T cells (Figure 5L-M). Moreover, T cells co-cultured with anti-ILT4- or anti-PD-L1-pretreated tumor cells secreted increased IFN-γ, and combination treatment most remarkably increased the release of IFN-γ into the supernatant (Figure 5N). More importantly, the cytolytic ability of T cells was significantly improved when they were co-cultured with anti-ILT4- or anti-PD-L1- or both antibody-pretreated tumor cells, in which the combination group generated the strongest tumor eradication in response to T cells (Figure 5O). Taken together, these results suggested that ILT4 blockade enhanced ICI activity in both TKI-resistant and EGFR wild-type NSCLC cells, affording a potential strategy to overcome ICI resistance in these patients.

### PIR-B and PD-L1 blockade synergistically prevented tumor growth and immune escape *in vivo*

**Figure 6:**
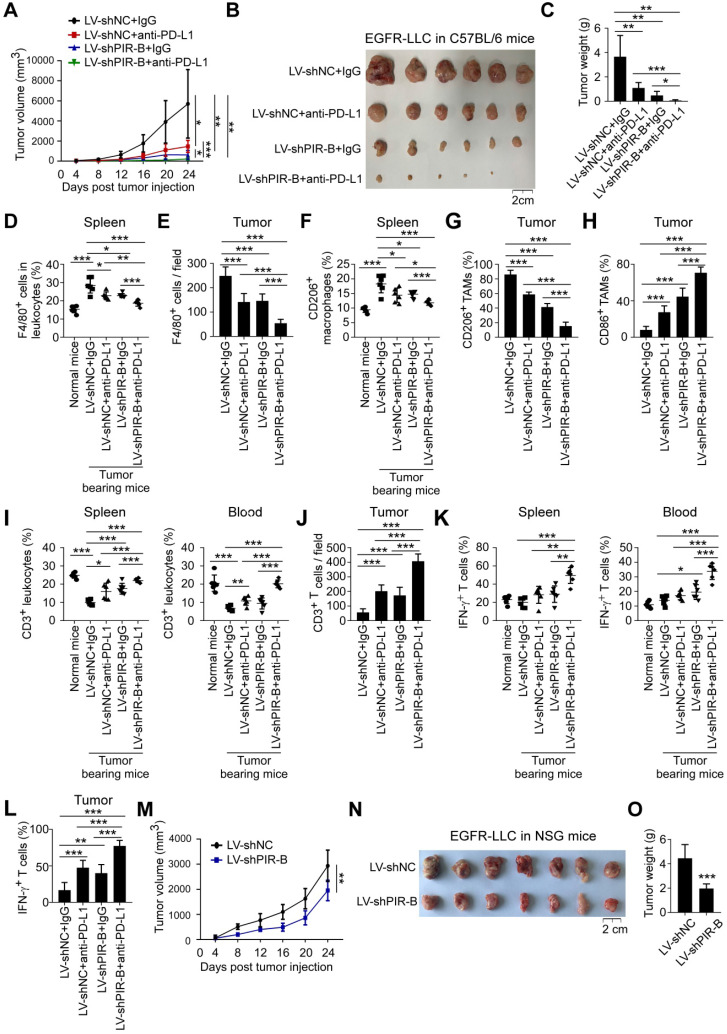
** PIR-B and PD-L1 blockade synergistically prevented tumor growth and immune escape *in vivo*. (A-B)** PIR-B knockdown or PD-L1 neutralization markedly inhibited transplanted tumor growth in C57BL/6 mice, while combined PIR-B and PD-L1 blockade yielded the slowest tumor growth rate. Mouse lung cancer cell line LLC (2×10^5^ cells/mouse) infected with both EGFR overexpression and PIR-B knockdown- or control lentivirus was subcutaneously inoculated into 6-8-week female C57BL/6 mice. PD-L1 inhibitory antibody or control IgG (200 µg) were intraperitoneally injected into the tumor-bearing mice every 4 days from day 7 post-tumor inoculation. Tumor sizes were measured every 4 days and presented as mean ± SD (n = 6 mice/group). (A) Tumor growth rate in each group and (B) Tumor images obtained from each mouse. Scale bar: 2 cm. **(C)** Tumors in PIR-B knockdown or PD-L1 inhibition groups showed significantly smaller tumor weight than the control group, while tumors in combined PIR-B and PD-L1 blockade groups showed the smallest tumor weight. The histogram shows the mean ± SD of tumor weights from each group at the endpoint of the experiments (n = 6 mice/group). **(D)** F4/80^+^ macrophages in CD45^+^leukocytes were remarkably increased in spleens of tumor-bearing mice compared with normal mice. However, when tumors were treated with PIR-B knockdown lentivirus or/and PD-L1 antibody, F4/80^+^ macrophages were markedly decreased with the lowest number in the combined PIR-B and PD-L1 blockade group. The proportions of F4/80^+^ macrophages in CD45^+^leukocytes were determined by flow cytometry. **(E)** Blocking PIR-B or PD-L1 remarkably decreased TAMs in tumor tissues, while combined PIR-B and PD-L1 blockade displayed the lowest number of TAMs. The accumulation of TAMs in mouse tumor tissues was evaluated by IHC analyses. **(F)** CD206^+^ macrophages in spleens were remarkably induced by tumor inoculation. However, when PIR-B or PD-L1 were blocked using specific knockdown lentivirus or neutralizing antibody, CD206^+^ macrophages were significantly reduced. The combined blockade of both molecules yielded the lowest CD206^+^ macrophage numbers in spleens. CD206^+^ macrophages in leukocytes were determined by flow cytometry. **(G-H)** PIR-B or PD-L1 inhibition markedly decreased CD206^+^ TAMs but increased CD86^+^ TAMs in transplanted tumor tissues, and combination therapy generated the lowest frequency of CD206^+^ TAMs but highest frequency of CD86+ TAMs in tumors. The accumulation of CD206^+^ TAMs (G) and CD86^+^ TAMs (H) was determined by IHC analysis.** (I)** The spleens and blood of tumor bearing mice showed much less CD3^+^ T cell proportion relative to normal mice. However, when PIR-B and/or PD-L1 were inhibited, the decreased proportion of CD3^+^ T cells in CD45^+^ lymphocytes was significantly reversed with the highest CD3^+^ T cell frequency in combined blockade group both in spleens and blood. The frequency of CD3^+^ T cells in CD45^+^ lymphocytes was detected using flow cytometry.** (J)** Tumor tissues from PIR-B-downregulated or anti-PD-L1-treated mice showed increased CD3^+^ T cell numbers in tumor tissues. Combined inhibition of both molecules generated the highest enrichment of CD3^+^ T cells as indicated by IHC analysis. **(K-L)** T cells in either PIR-B or PD-L1 blockade group displayed increased IFN-γ expression, while the combination therapy group showed the highest IFN-γ levels. IFN-γ expression in mouse spleens and blood (K) was determined by flow cytometry and in tumors (L) by IHC. **(M-O)** Knockdown of PIR-B in LLC inhibited tumor growth, and decreased tumor sizes and weights in immunodeficient NSG mice. Cell preparation, cell injection numbers and procedures were identical as described in (A). Tumor sizes were measured every 4 days (n = 7 mice/group). (M) Tumor growth rate in each group and (N) Tumor images from each mouse. Scale bar: 2 cm. (O) Tumors in PIR-B knockdown group showed smaller tumor weight than the control group. Data in (M) and (O) are presented as mean ± SD. *, *p* < 0.05; **, *p* < 0.01; ***, *p* < 0.001. Anti: inhibitory antibody; LV-shPIR-B: lentivirus carrying PIR-B shRNA; LV-shNC: lentivirus carrying control shRNA.

Our *in vitro* studies indicated that ILT4 led to the infiltration of M2-like TAMs and hypo-responsiveness of T cells, and blockade of ILT4 significantly curtailed these effects and enhanced the activity of the PD-L1 inhibitor. We next explored whether combined ILT4 and PD-L1 blockade had a synergistic effect on controlling tumor development, M2-like TAM infiltration, and T cell dysfunction* in vivo*. LLC mouse lung cancer cells were first transfected with EGFR-containing lentiviruses to activate EGFR signaling. Then, PIR-B (ILT4 orthologue in mouse) knockdown or control lentiviruses were transfected into EGFR-upregulated LLC cells (EGFR-LLC). As displayed in Figure S6A-B, EGFR overexpression activated EGFR phosphorylation and PIR-B expression, while PIR-B knockdown decreased PIR-B rather than pEGFR levels. We subcutaneously inoculated 2×10^5^ PIR-B-downregulated EGFR-LLC cells into wild-type C57BL/6 mice. After 7 days, anti-PD-L1 or control IgG were intraperitoneally injected into tumor-bearing mice every 4 days, and the tumor sizes were measured. The efficiency of PIR-B knockdown was also confirmed in transplanted tumor tissues (Figure S6C). Figure 6A shows that both PIR-B knockdown and PD-L1 blockade slowed down tumor growth compared with the control group, while combined blockade of both molecules displayed a cooperative effect on tumor inhibition. We confirmed these results by measuring final tumor sizes and tumor weights (Figure 6B-C). Next, we determined whether PIR-B and PD-L1 blockade reprogrammed the infiltration and functionality of TAMs and T cells. Immunocytes from mouse spleens and blood were separated and the quantities, phenotypes, and subsets of TAMs and T cells were analyzed by flow cytometry. The infiltration and phenotypes of TAMs and T cells in tumor tissues were assessed by IHC analysis. We found a significant accumulation of F4/80^+^ macrophages in spleens of tumor-bearing mice compared with normal tumor-free mice (Figure 6D). However, blockade of either PIR-B or PD-L1 partially prevented TAMs infiltration in spleens, and combined blockade by both almost completely inhibited TAM accumulation (Figure 6D). Similarly, inhibition of PIR-B or PD-L1 or both markedly decreased TAM density in tumor tissues with the combination group displaying the most significant decrease in TAM density (Figure 6E, Figure S6D-E). We also analyzed the M2-like phenotype of TAMs by detecting CD206-positive cells in spleens and CD206/CD163/CD86/CD80-positive cells in tumor tissues. As expected, tumor-bearing mice showed more frequent CD206^+^ macrophages in spleens than tumor-free mice, whereas PIR-B and PD-L1 blockade alone or in combination markedly decreased the frequency of CD206^+^ macrophages with the most apparent decrease in the combined blockade group (Figure 6F). Similarly, PIR-B or PD-L1 inhibition reduced the proportion of CD206^+^ and CD163^+^ TAMs but elevated that of CD86^+^ and CD80^+^ TAMs in tumor tissues with the most obvious alteration in the combined blockade group (Figure 6G-H, Figure S6D-G).

Besides the impact on TAM recruitment and polarization, the accumulation of T cells in spleens and blood was inhibited in tumor-inoculated mice compared with normal mice (Figure 6I). However, blockade of either PIR-B or PD-L1 in tumors partially restored T cell infiltration and combined blockade almost totally restored T cell accumulation in spleens, blood, and tumor tissues (Figure 6I-J, Figure S6H). Furthermore, PIR-B and PD-L1 blockade alone or in combination improved IFN-γ levels in T cells from spleens, blood, and tumor tissues, with the most noticeable improvement in the combination group (Figure 6K-L, Figure S6H). Since CD4^+^ and CD8^+^ T subsets represent two major populations comprising more than 90% of total T cells 33, we next determined the effect of PIR-B and PD-L1 blockade on T cell subset distribution. Compared with normal mice, tumor-bearing mice showed distinctly reduced CD4^+^ T cell subset and enhanced CD8^+^ T cell subset frequencies in both spleens and blood (Figure S6I-J). However, neither PIR-B nor PD-L1 inhibition affected T cell subset distribution in spleens and blood of tumor-bearing mice compared with the control group (Figure S6I-J). We also analyzed the production of IFN-γ by different T cell subsets. As is evident from Figure S6K-L, in both CD4^+^ T and CD8^+^ T cell subtypes, PIR-B- and PD-L1-modulated IFN-γ alterations were similar to those observed in the total T cell population, suggesting a subset-independent control of PIR-B and PD-L1 of T cell infiltration and function.

To further dissect the importance of PIR-B on anti-tumor immunity, we transplanted the same EGFR-LLC cells into immunodeficient NSG mice to evaluate its effect on tumor biology. We found that knockdown of PIR-B slowed down tumor growth compared with the control group (Figure 6M-O). However, the intergroup difference was much smaller than that observed in wild-type C57BL/6 mice (Figure 6A-C), suggesting that PIR-B-mediated immunosuppression participated in tumor progression. Collectively, these results indicated that PIR-B and PD-L1 blockade synergistically normalized the immunosuppressive TME and prevented tumor growth and immune escape *in vivo*, rationalizing the combination of ILT4 and PD-L1 blockade in lung cancer treatment.

### ILT4 blockade alone rather than in combination with PD-L1 inhibitor suppressed tumor progression and immune evasion in TKI-resistant EGFR mutant NSCLC *in vivo*

**Figure 7:**
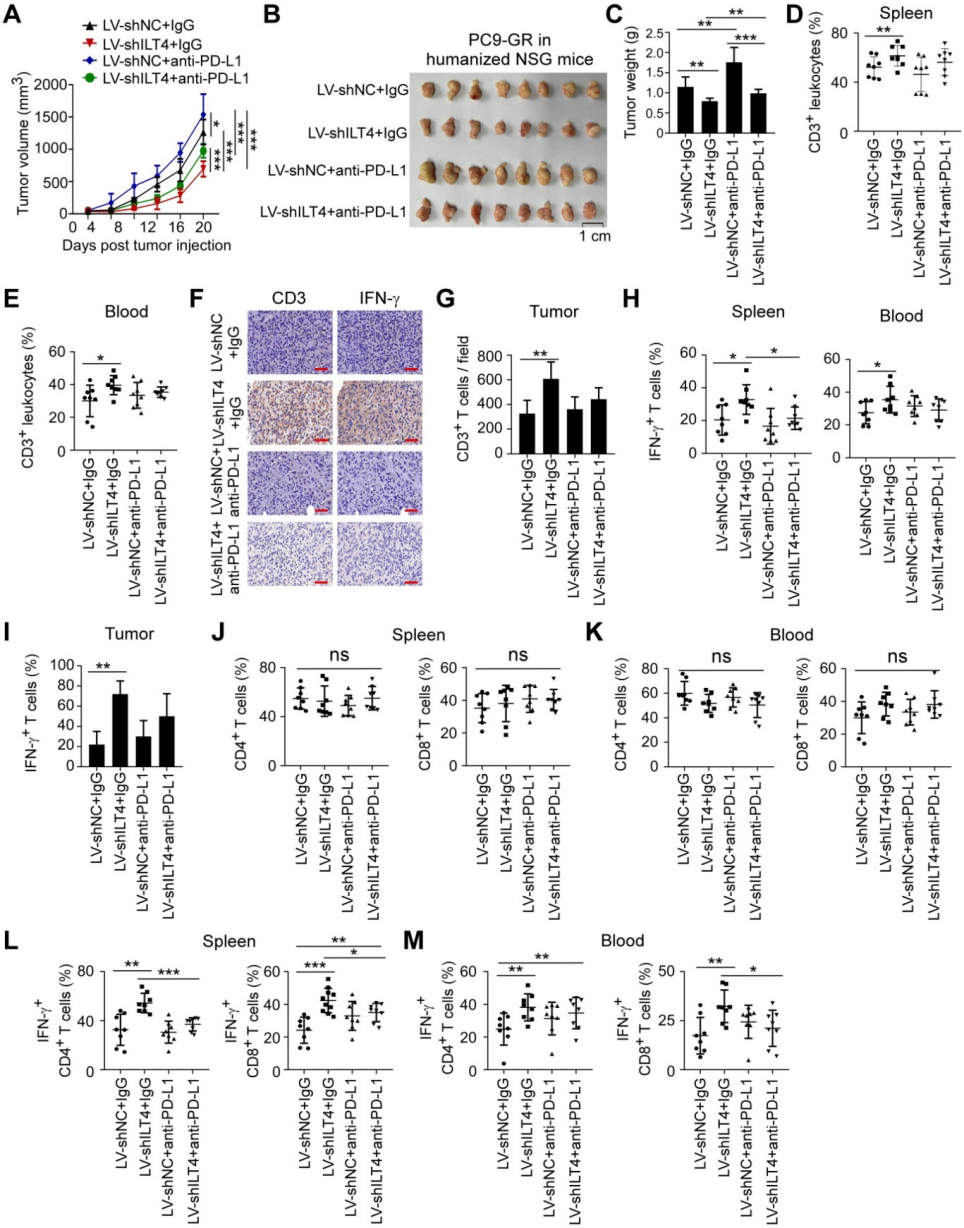
** ILT4 blockade suppressed tumor progression and immune escape in TKI-resistant EGFR mutant NSCLC *in vivo*. (A-B)** ILT4 knockdown markedly inhibited PC9-GR growth in humanized NSG mice. However, PD-L1 blockade accelerated PC9-GR growth and combined blockade of both molecules showed no tumor inhibition compared to the control group. PC9-GR cells (3×10^6^ cells/mouse) transfected with lentiviruses carrying ILT4-knockdown or control shRNA were subcutaneously injected into 6-8-week female NSG mice. Day 7 after tumor inoculation, 2×10^7^ PBMCs from healthy volunteers were transplanted into each mouse via the tail vein. Anti-PD-L1 or control IgG (200 µg/mouse) were intraperitoneally injected into the tumor-bearing mice every 4 days from the day of PBMC transplant. Tumor sizes were measured every 4 days and presented as mean ± SD (n = 8 mice/group). (A) Tumor growth rate in each group and (B) Tumor images from each mouse. Scale bar: 1cm. **(C)** Tumors in ILT4 knockdown group showed significantly smaller tumor weight than in the control group. However, PD-L1 blockade led to higher while combined blockade generated similar tumor weight compared with the control group. The histogram shows the mean ± SD of tumor weights from each group at the experiment endpoint (n = 8 mice/group). **(D-E)** ILT4 knockdown increased CD3^+^ T cells in leukocytes in both spleens (D) and blood (E) of tumor-bearing mice. However, PD-L1 blockade alone or in combination with ILT4 inhibition did not affect CD3^+^ T cells in these organs. The frequency of CD3^+^ T cells in tumor spleens and blood were determined by flow cytometry.** (F-G)** ILT4 knockdown promoted the infiltration of CD3^+^ T cells in tumors by IHC staining. However, PD-L1 blockade alone or in combination with ILT4 inhibition did not alter the number of CD3^+^ T cells in tumor tissues. (F) Images of T cell infiltrates and (G) Average results from 8 mice in each group. Scale bar: 20 µm. **(H-I)** The IFN-γ levels in CD3^+^ T cells from blood, spleens, and tumors were remarkably increased by ILT4 knockdown compared with PD-L1 blockade or combined ILT4 and PD-L1 blockade. (H) The IFN-γ expression in blood and spleens were analyzed by flow cytometry, and (I) in tumors by IHC. **(J-K)** The distribution of CD4^+^ and CD8^+^ T cell subsets in mouse blood (J) and spleens (K) was not altered by inhibition of ILT4 or PD-L1 or both molecules as determined by flow cytometry analysis. **(L-M)** The IFN-γ expression in both CD4^+^ and CD8^+^ T cells was augmented by ILT4 knockdown but not by anti-PD-L1 or combination therapy. Results are shown in spleens (L) and blood (M). The level of IFN-γ expression in both organs was evaluated by flow cytometry. *, *p* < 0.05; **, *p* < 0.01; ***, *p* < 0.001. LV-shILT4: lentivirus carrying ILT4 shRNA; LV-shNC: lentivirus carrying control shRNA; ns: no significance.

For NSCLC patients with TKI-resistant EGFR mutant, ICI resistance is still a challenge 6. Based on the above results, we speculated that ILT4 blockade might be effective in overcoming the ICI resistance. We first established an immunotherapy model in immune-reconstructed NSG mice using the TKI-resistant EGFR mutant NSCLC cell line PC9-GR. We subcutaneously inoculated 6-8-week-old NSG mice with 2×10^6^ PC9-GR cells transfected with ILT4 knockdown lentivirus. The efficiency of ILT4 knockdown in PC9-GR cells was confirmed at both mRNA and protein levels before tumor implantation (Figure S6M-N). On day 7 after tumor transplantation, 2×10^7^ PBMCs from healthy volunteers were injected into each mouse via the tail vein. Anti-PD-L1 was intraperitoneally injected into tumor-bearing mice every 4 days from the 7th day after tumor inoculation and tumor sizes were measured simultaneously. Tumor growth in the ILT4 knockdown group was much slower than in the control group (Figure 7A). Surprisingly, PD-L1 blockade promoted rather than inhibited tumor growth in PC9-GR control and ILT4 knockdown groups (Figure 7A). ILT4 blockade could not reverse ICI resistance in this subpopulation. The final tumor sizes and weights also yielded consistent results (Figure 7B-C). The ILT4- and PD-L1-regulated T cell immunity was investigated by separating T cells from mouse spleens and blood and detecting T cell abundance and phenotypes in different groups using flow cytometry. The T cell infiltration and phenotypes in tumor tissues were examined by IHC staining. As expected, ILT4 knockdown markedly elevated T cell infiltration in spleens (Figure 7D), blood (Figure 7E) and tumor tissues (Figure 7F-G). However, neither PD-L1 blockade alone nor in combination with ILT4 inhibition affected T cell accumulation in these organs (Figure 7D-G). Also, T cells in the ILT4 knockdown group rather than in the PD-L1 blockade or combination therapy group displayed increased IFN-γ levels in blood, spleens and tumor tissues compared with the control group (Figure 7H-I). Consistent with the results obtained in C56BL/6 mice, ILT4 inhibition did not alter the T subset distribution in both spleens and blood (Figure 7J-K). Furthermore, ILT4 blockade augmented IFN-γ expression in both CD4^+^ and CD8^+^ T cell subsets from mouse blood and spleens (Figure 7L-M). These results clearly indicated that ILT4 blockade might be an effective treatment strategy for EGFR mutant patients resistant to EGFR-TKI treatment.

### ILT4 blockade enhanced the efficacy of PD-L1 inhibitor in EGFR wild-type NSCLC *in vivo*

ICI therapy has been applied to EGFR wild-type NSCLC patients in most clinical trials 34. However, the response rate of single-agent ICIs in these patients was moderate and no more than 20% 4, indicating that most patients were not sensitive to ICI treatment. Improving the efficacy of ICIs in this subpopulation is a clinical challenge in NSCLC treatment. Since combined blockade of ILT4 and PD-L1 showed a synergistic effect on tumor inhibition *in vitro*, we explored whether combination therapy could increase ICI treatment potential in EGFR wild-type NSCLC *in vivo*.

**Figure 8:**
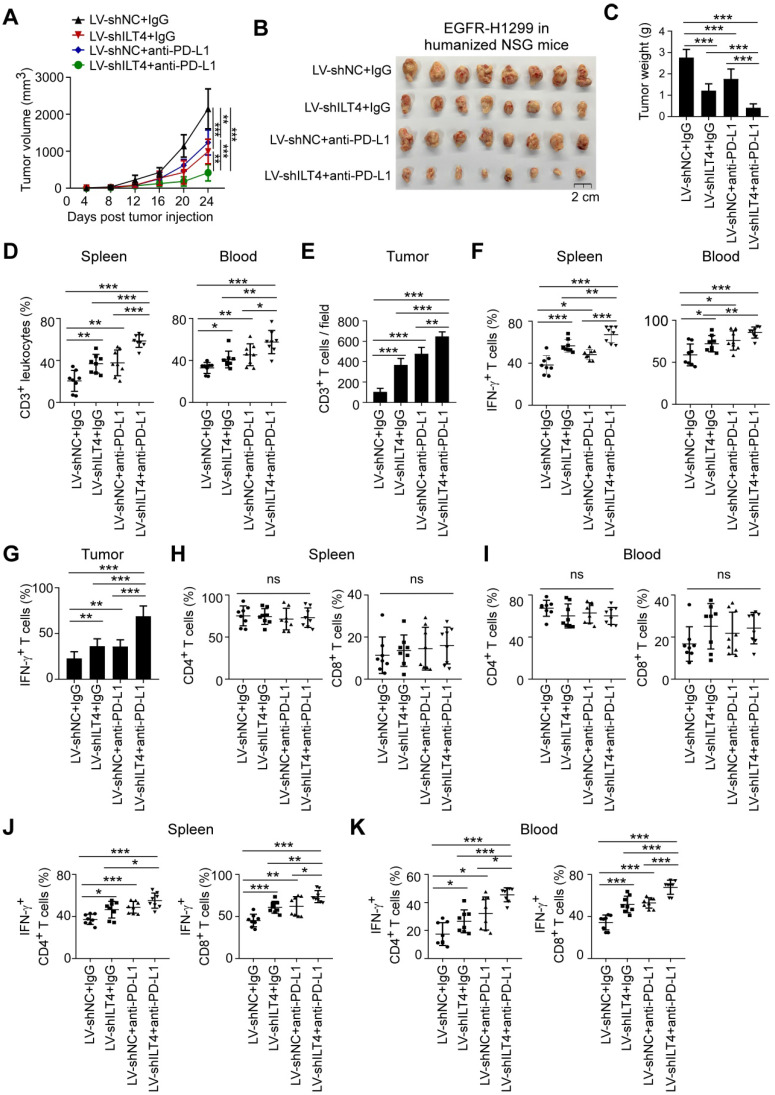
** ILT4 blockade enhanced the efficacy of PD-L1 inhibitor in EGFR wild-type NSCLC *in vivo*. (A-B)** ILT4 or PD-L1 blockade markedly slowed H1299 growth in humanized NSG mice compared with the control group, and combination treatment generated the lowest tumor growth rate. EGFR-overexpressing H1299 cells (3×10^6^ cells/mouse) were first transfected with ILT4 knockdown or control lentiviruses, and then inoculated into the right flanks of 6-8-week female NSG mice. On day 7 post tumor inoculation, 2×10^7^ PBMCs from healthy volunteers were intravenously injected into each mouse. Anti-PD-L1 or control IgG (200 µg/mouse) were intraperitoneally injected into the tumor-bearing mice every 4 days from the day of PBMC transplant. Tumor sizes were measured every 4 days and presented as mean ± SD (n = 8 mice/group). (A) Tumor growth rate in each group and (B) Tumor images for each mouse. Scale bar: 2 cm. **(C)** Tumors in ILT4 or PD-L1 blockade group showed significantly smaller tumor weight than the control group, while combination therapy showed synergy in tumor inhibition. The histogram shows the mean ± SD of tumor weights from each group at the experiment endpoint (n = 8 mice/group). **(D-E)** Spleen, blood and tumor tissues in ILT4 or PD-L1 blockade group showed significantly higher CD3^+^ T cell frequency than the control group, while the combination therapy group showed the highest CD3^+^ T cell infiltrates. (D) The CD3^+^ T cell frequency in mouse spleen and blood was detected by flow cytometry and (E) tumors were detected by IHC respectively. **(F-G)** ILT4 or PD-L1 blockade increased IFN-γ expression in T cells of spleen, blood and tumors relative to the control group, and combination therapy yielded the highest IFN-γ in T cells. The IFN-γ expression in mouse spleen, blood and tumors was detected by flow cytometry and IHC, respectively. **(H-I)** The distribution of CD4^+^ and CD8^+^ T cell subsets in spleen (H) and blood (I) was not altered by single or combination blockade of ILT4 and PD-L1 as determined by flow cytometry **(J-K)** ILT4 or PD-L1 blockade increased IFN-γ expression in CD4^+^ and CD8^+^ T cells of spleen (J) and blood (K) with the highest IFN-γ level in combination therapy group by flow cytometry analysis. *, *p* < 0.05; **, *p* < 0.01; ***, *p* < 0.001. LV-shILT4: lentivirus carrying ILT4 shRNA; LV-shNC: lentivirus carrying control shRNA; ns: no significance.

The immunotherapeutic models were established using EGFR-overexpressing and ILT4-downregulated H1299 cells and the immune-reconstructed NSG mice following the procedures described above. The efficiency of ILT4 knockdown in EGFR-H1299 cells was validated at both mRNA and protein levels before tumor injection (Figure S6O-P). Tumor sizes were measured every 4 days post tumor transplant. Consistent with the *in vitro* results, either ILT4 or PD-L1 blockade decreased tumor growth *in vivo*, and combination therapy most significantly suppressed tumor growth compared with any other group (Figure 8A-C). To clarify the regulation of combined ILT4 and PD-L1 inhibition in T cell immunity, we analyzed T cell infiltration in spleens, blood, and tumor tissues of tumor-bearing mice. As displayed in Figure 8D-E, either ILT4 or PD-L1 inhibition promoted T cell accumulation in these organs compared with the control group, while combination therapy yielded the highest T cell accumulation. Moreover, IFN-γ produced by these T cells was also markedly elevated by ILT4 and/or PD-L1 blockade and the combination blockade generated the highest IFN-γ levels in spleens, blood and tumor tissues (Figure 8F-G). Likewise, T cell subset distribution in mouse spleens and blood was not altered by any of the treatment (Figure 8H-I), and the treatment-altered IFN-γ levels in both CD4^+^ and CD8^+^ T cell subsets were similar to those in total T cells (Figure 8J-K). These results collectively revealed that ILT4 and PD-L1 blockade synergistically cooperated to inhibit tumor growth and immune evasion. Thus, ILT4 blockade appears to be a desirable strategy to improve ICI efficacy in NSCLC patients with wild-type and activated EGFR.

## Discussion

ICI therapy has greatly changed the paradigm of NSCLC treatment 34. However, its efficacy in EGFR wild-type patients is less than 20% 3, 4, while the response rate in EGFR-driven subpopulation is even poorer and mostly invalid 6. Given that EGFR in most NSCLC patients is activated 35, restricting anti-tumor immune response and ICI efficacy 6, identification of the underlying mechanisms for EGFR-mediated immunosuppression and reshaping the tumoricidal immune microenvironment is urgently needed to maximize the clinical benefit of ICIs in EGFR-activated NSCLC. Previous studies from our laboratory and other groups have suggested ILT4 as a potential checkpoint for tumor immunotherapy 10, 15. In the current study, we found that ILT4 expression in NSCLC cells could be induced by activated EGFR-AKT/-ERK1/2 signaling. ILT4 in EGFR-activated NSCLC cells induced TAM recruitment and M2-like polarization, inhibited T cell infiltration and cytotoxicity, and consequently created an immunosuppressive and tumor-promoting TME. ILT4 blockade or knockdown prevented immunosuppression and tumor progression both *in vitro* and *in vivo*. Significantly, using humanized murine immunotherapy models, we established ILT4 inhibition as an attractive approach for TKI-resistant EGFR-mutant NSCLC patients. Furthermore, ILT4 inhibition substantially potentiated the anti-tumor activity of PD-L1 inhibitor in the EGFR wild-type NSCLC subtype.

ILT4 is an inhibitory receptor expressed in myeloid cells, including dendritic cells (DCs), macrophages, neutrophils, and platelets 9, 10. ILT4 in these cells inhibited DC maturation and antigen presentation, reprogrammed the M2-like phenotype of macrophages, impaired the phagocytic ability of neutrophils and restricted the aggregation of platelets 10. Induced ILT4 expression in activated T cells was reported to drive the differentiation of Th2 subsets 36. In the last decade, we and others identified ILT4 expression in multiple solid tumors, promoting tumor cell proliferation, invasion, and metastasis 10, 11, 13, 37. Meanwhile, high ILT4 levels in tumor cells predicted decreased T cell infiltration and inhibitory T cell subset distribution in lung cancer tissues 11, 15, suggesting that ILT4 also participated in tumor immune escape. However, the exact regulation of tumor cell ILT4 to immune microenvironment is still undetermined.

Here, we reported, for the first time, that ILT4 suppressed T cell immunity directly by impairing T cell activity and response, and indirectly through recruitment of M2-like TAMs, the most abundant immune cell population in the TME 38. During the initiation and evolution of malignancies, circulating monocyte precursors are recruited into the TME and then polarized into pro-tumoral M2-like TAMs, which exhibit tumor-promoting activity directly controlling tumor cell growth and invasion, and indirectly through inducing tumor angiogenesis and immunosuppression 20, 39. Therefore, accumulation and M2-like polarization of TAMs represent two key steps in TAM-mediated tumor promotion. A better understanding of these processes has led to the development of multiple strategies modulating TAM recruitment, survival, and polarization 38. However, these agents are still in the early stages of clinical trials and merely generated mild benefit 38. A deeper understanding of TAM recruitment and polarization mechanisms, and designing efficacious combination therapies are future directions to maximize their anti-tumor efficacy.

The causative link between ILT4 expression and the accumulation of pro-tumoral TAMs we identified has expanded the mechanisms for tumor cell-remodeled TAM function in tumor promotion. We also found that ILT4 regulated the secretion of classic chemokines CCL2 and CCL5, which are responsible for TAM recruitment into the TME. These results underscore the importance of chemokine inhibition in reversal of TAM-impaired tumor immunity 40. Surprisingly, other canonical drivers for macrophage attraction including CSF-1, CCL8 and CXCLs was not consistently regulated by ILT4, reflecting the intercellular heterogeneity, and complex mechanisms in maintaining cell homeostasis 41.

Besides regulating TAMs, ILT4 in tumor cells impeded the proliferation and killing ability of T cells. Effector T cells are the most potent weapons for tumor eradication 42. T cell accumulation in the tumor bed is considered a predictor of superior patient outcomes 43. However, tumor cells can utilize multiple strategies, including recruitment of inhibitory cells, production of suppressive cytokines and chemokines, metabolic competition, and expression of inhibitory molecules, to restrict T cell infiltration and immune response 44. Here we found that ILT4 overexpression is an important mechanism for T cell exclusion and dysfunction, posing a direct obstacle to anti-tumor immunity. Based on our results, it is plausible that blocking ILT4 might prevent TAM-mediated immunosuppression, improve infiltration and bioactivity of tumor-specific T cells, and break the hostile immune barrier in TME. Indeed, our *in vitro* and *in vivo* studies have demonstrated that ILT4 blockade decreased M2-like TAM accumulation and restored infiltration and tumoricidal function of T cells, thus inhibiting tumor growth.

EGFR mutations are the most common oncogenic drivers for NSCLC initiation and progression 45. Activation of EGFR and the downstream signaling network impacts multiple cellular processes including proliferation, survival, invasion, and metastasis, facilitating tumor progression 46. Activated EGFR signaling also induces the immunosuppressive TME through recruiting or reprogramming suppressive immunocytes, inhibiting MHC molecule levels, and upregulating inhibitory cytokines and metabolites 6. In our current study, EGFR activation induced aberrant expression of ILT4, a novel mechanism for EGFR-induced immunosuppression. There are two distinct mechanisms to activate EGFR in cancer cells. One is ligand-dependent activation induced by engagement of EGF, TGF-α or amphiregulin, and the other is constitutive activation caused by tyrosine kinase mutation in EGFR 46. We observed that in both cases, activated EGFR upregulated ILT4 expression and led to immunosuppression, suggesting that ILT4 blockade is pertinent in patients with EGFR mutant and wild-type forms. Our *in vivo* study also verified that in both EGFR mutant and EGF-activated EGFR wild-type tumors, ILT4 inhibition was an appropriate approach for treating NSCLC. Our results also illustrated that EGFR-upregulated ILT4 expression was mediated through MAPK/ERK and PI3K/AKT signaling. However, the mechanisms for EGFR activation-induced ILT4 expression need further investigation.

Specific inhibition of EGFR activity using EGFR-TKIs has become the preferred first-line therapy in NSCLC patients harboring EGFR mutation since the last decade 31, 47. However, acquired resistance to first- or new-generation EGFR-TKIs invariably develops, resulting in inevitable disease progression 48-50. Treatment options are limited for these patients due to severe adverse effects or poor response rates 32. Despite the remarkable success of ICIs in patients without driver oncogene alteration, the efficacities and the optimal use of ICIs in EGFR-driven tumors are still controversial and largely unsatisfying 51, underscoring the need to explore novel immuno-therapeutics for this subpopulation. Our present study found that ILT4 inhibition reconfigured the immunosuppressive and tumor-promoting microenvironment, and repressed the progression of EGFR mutant NSCLC. Using a humanized immunotherapy model of EGFR-resistant NSCLC, we elucidated that ILT4 inhibition could be used as the second-line therapy in EGFR mutant patients who acquire resistance to EGFR-TKI treatment.

Since immunosuppressive TME, one of the major causes for ICI resistance in EGFR mutant patients, could be partially reversed by ILT4 inhibition, we speculated that ILT4 blockade might be effective to overcome the primary hypo-responsiveness of ICIs in this NSCLC subpopulation. Unexpectedly, although we obtained positive results in *in vitro* studies, combining ILT4 inhibition with anti-PD-L1 therapy did not improve its efficacy *in vivo*. On the contrary, treatment with PD-L1 inhibitor yielded larger tumor sizes compared with the control group. This observation was consistent with the results reported by a panel of clinical and preclinical studies where EGFR mutations were suggested as a genomic biomarker of increased hyper-progression risk 52. Meanwhile, these results implied that the TME is too complicated to be simulated in the *in vitro* system. More importantly, this controversy highlighted that EGFR-manipulated ICI resistance mechanisms are far more complex than simple immunosuppression mediated by TAMs and dysfunctional T cells. Other escape routes like MHC inhibition, low TMB, Tregs, tolerated DCs and myeloid-derived suppressor cells (MDSCs) might play more pivotal roles in this process 6, 53. However, all these issues warrant in-depth investigation and validation.

ICIs are currently recommended as the first-line management in metastatic EGFR wild-type NSCLC 34. However, the response rate is moderate and no more than 20% 3, 4. Improvement in clinical benefits relies developing different combination strategies, including chemotherapy, radiotherapy, targeted therapy, and immunotherapy 54. Despite remarkable advances in combined ICIs and chemotherapies, combination immunotherapy is of greater clinical importance due to low side effects 55. The combination of different ICIs with complementary mechanisms of action has been widely investigated in NSCLC 56, however, the clinical benefit is still unsatisfactory. Therefore, the development of novel combination regimens in the EGFR wild-type subpopulation is urgently needed. Herein, using a humanized murine immunotherapy model, we demonstrated that ILT4 antagonism enhanced the efficacy of PD-L1 inhibitor in EGFR wild-type NSCLC. As reported previously 6, the suppressive immune microenvironment is a major hurdle to effective anti-tumor immunotherapy. In the current study, we found that ILT4 overexpression led to the immunosuppressive TME and tumor promotion in EGFR wild-type NSCLC, rationalizing the synergy of combined ILT4 inhibition with ICI treatment. Furthermore, increased ILT4 and PD-L1 co-expression in human NSCLC tissues with wild-type EGFR suggested that the combination blockade of ILT4 and PD-L1 is clinically feasible for a broad spectrum of NSCLC patients.

## Conclusion

In this study, we provided evidence that activated EGFR signaling induced ILT4 overexpression in NSCLC cells via ERK1/2 and AKT signaling pathways. We also elucidated novel mechanisms for ILT4-mediated tumor immune escape in EGFR-activated NSCLC, involving recruitment of pro-tumor TAMs and inhibition of T cell immunity. Significantly, we demonstrated that ILT4 inhibition reversed the immunosuppressive TME and might be a promising strategy for the second-line treatment of TKI-resistant EGFR-mutant NSCLC. Furthermore, we established an attractive combination approach of ILT4 inhibition with ICIs for EGFR wild-type NSCLC (Figure S7).

## Supplementary Material

Supplementary figures and tables.

## Figures and Tables

**Table 1:** Correlation between pEGFR/ILT4 co-expression and clinicopathological characteristics in human NSCLC (n = 80)

Variables	pEGFR+	pEGFR-	*p* value	pEGFR-	*p* value	pEGFR+	*p* value
	ILT4+	ILT4-		ILT4+		ILT4-	
**Age (years)**							
≤ 60	8	4	0.8253	6	0.2258	6	0.3691
> 60	24	14		8		10	
**Sex**							
Male	24	6	0.0039	8	0.2258	10	0.3691
Female	8	12		6		6	
**Histological types**							
ADC	20	16	0.0461	8	0.7319	8	0.4076
SCC	12	2		6		8	
**EGFR mutation**							
Yes	14	12	0.7393	6	0.7913	6	0.7913
No	6	4		2		2	
**Differentiation**							
Well/moderate	11	10	0.1452	8	0.149	11	0.0242
Poor	21	8		6		5	
**Primary tumor size**							
≤ 5cm	24	16	0.2386	6	0.0352	12	0.999
> 5cm	8	2		8		4	
**Lymph node involvement**							
Yes	18	10	0.9621	8	0.9552	10	0.6788
No	14	8		6		6	
**Pleural metastasis**							
Yes	20	6	0.0475	12	0.1154	4	0.0143
No	12	12		2		12	
**TNM stages**							
I-II	16	8	0.7059	6	0.6554	10	0.4126
III-IV	16	10		8		6	
**PD-L1 expression**							
Negative	8	15	0.0001	2	0.3148	9	0.0328
Positive	24	3		14		7	

ADC: adenocarcinoma; SCC: squamous cell carcinoma. Compared all other three groups with pEGFR+ILT4+ group.

## Abbreviations

ADC: adenocarcinoma
CCL: chemokine (C-C motif) ligand
CXCL: chemokine (C-X-C motif) ligand
CM: conditioned medium
CSF: colony stimulating factor
CTL: cytotoxic T lymphocyte
DAB: 3,3′-diaminobenzidine solution
DC: dendritic cell
E:T ratio: effector target cell ratio
EGF: epidermal growth factor
EGF-H1299: EGF-preactivated H1299
EGFR: epidermal growth factor receptor
ELISA: enzyme linked immunosorbent assay
FBS: fetal bovine serum
HRP: streptavidin-conjugated horseradish peroxidase
ICI: immune checkpoint inhibitor
IHC: immunohistochemistry
ILT4: immunoglobulin-like transcript 4
KEGG: Kyoto Encyclopedia of Genes and Genomes
LDH: lactate dehydrogenase
LLC: Lewis lung carcinoma
M-CSF: macrophage colony stimulating factor
MDSC: myeloid-derived suppressor cell
MHC: major histocompatibility complex
NSCLC: non-small cell lung cancer
PBMC: peripheral blood mononuclear cell
PC9-GR: gefitinib-resistant PC9
pEGFR: phosphorylated EGFR
PIR-B: paired Ig-like receptor B
SCC: squamous cell carcinoma
siRNA: small interfering RNA
TAM: tumor-associated macrophage
TCGA: The Cancer Genome Atlas
TKI: tyrosine kinase inhibitor
TMB: tumor mutation burden
TME: tumor microenvironment
Treg: regulatory T cell
