# Supplementary material for: ILT4 inhibition prevents TAM- and dysfunctional T cell-mediated immunosuppression and enhances the efficacy of anti-PD-L1 therapy in NSCLC with EGFR activation
Source: Theranostics. 2021 Jan 19;11(7):3392–416. doi: 10.7150/thno.52435 (PMC7847666; doi:10.7150/thno.52435)
Supplement: Supplementary file 1 — Supplementary figures and tables. [file thnov11p3392s1.pdf]

Supplementary table 1: Specific primers used in this work

Target genes	Forward	Reverse
**Human**		
ILT4	ACCCAAAACCCACCCTCTC	ACAGAATGAAGCCGCCAAA
EGFR	ATCATACGCGGCAGGACCA	TCTGACCGGAGGTCCCAAAC
EGF	CGAGCAGAGATGTGAGGAGTC	CATTCTTGAGGTCTTGGTGTTCTT
IL-6	AAGCCAGAGCTGTGCAGATGAGTA	TGTCCTGCAGCCACTGGTTC
ARG-1	CTGGCAAGGTGGCAGAAGTC	ATGGCCAGAGATGCTTCCAA
IL-12	AGGGCCGTCAGCAACATG	TCTTCAGAAGTGCAAGGGTAAAATTC
TNF-α	CAGAGGGAAGAGTTCCCCAG	CCTGGTCTGGTAGGAGACG
IL-10	GCTGTCATCGATTTCTTCCC	CTCATGGCTTTGTAGATGCCT
CCL2	CTTCTGTGCCTGCTGCTCATA	CTTTGGGACACTTGCTGCTG
CCL5	ACCAGTGGCAAGTGCTCCAAC	CAGCCGGGAGTCATACAGGAA
CCL8	TGGAGAGCTACACAAGAATCACC	TGGTCC AGATGCTTCATGGAA
CXCL1	CAAACCGAAGTCATAGCCACAC	GGATTTGTCACTGTTCAGCATCTT
CXCL9	TCTTGCTGGTTCTGATTGGAGTG	TAGTCCCTTGGTTGGTGCTGAT
CXCL10	GGCCATCAAGAATTTACTGAAAGCA	TCTGTGTGGTCCATCCTTGGAA
CXCL11	CCTTGGCTGTGATATTGTGTGCTA	CCTATGCAAAGACAGCGTCCTC
CSF1	GATGGAGACCTCGTGCCAAATTA	TGTTATCTCTGAAGCGCATGGTG
GAPDH	GCACCGTCAAGGCTGAGAAC	TGGTGAAGACGCCAGTGGA
**Mouse**		
EGFR	GCCATCTGGGCCAAAGATACC	GTCTTCGCATGAATAGGCCAAT
PIR-B	ACCCAGGAAGAAAGCCTATATG	GTTCAGTTGTTCCCTTGACATG
PD-L1	GCTCCAAAGGACTTGTACGTG	TGATCTGAAGGGCAGCATTTC
GAPDH	ATGAAGGGGTCGTTGATGGC	GGGTTCCTATAAATACGGACTGC

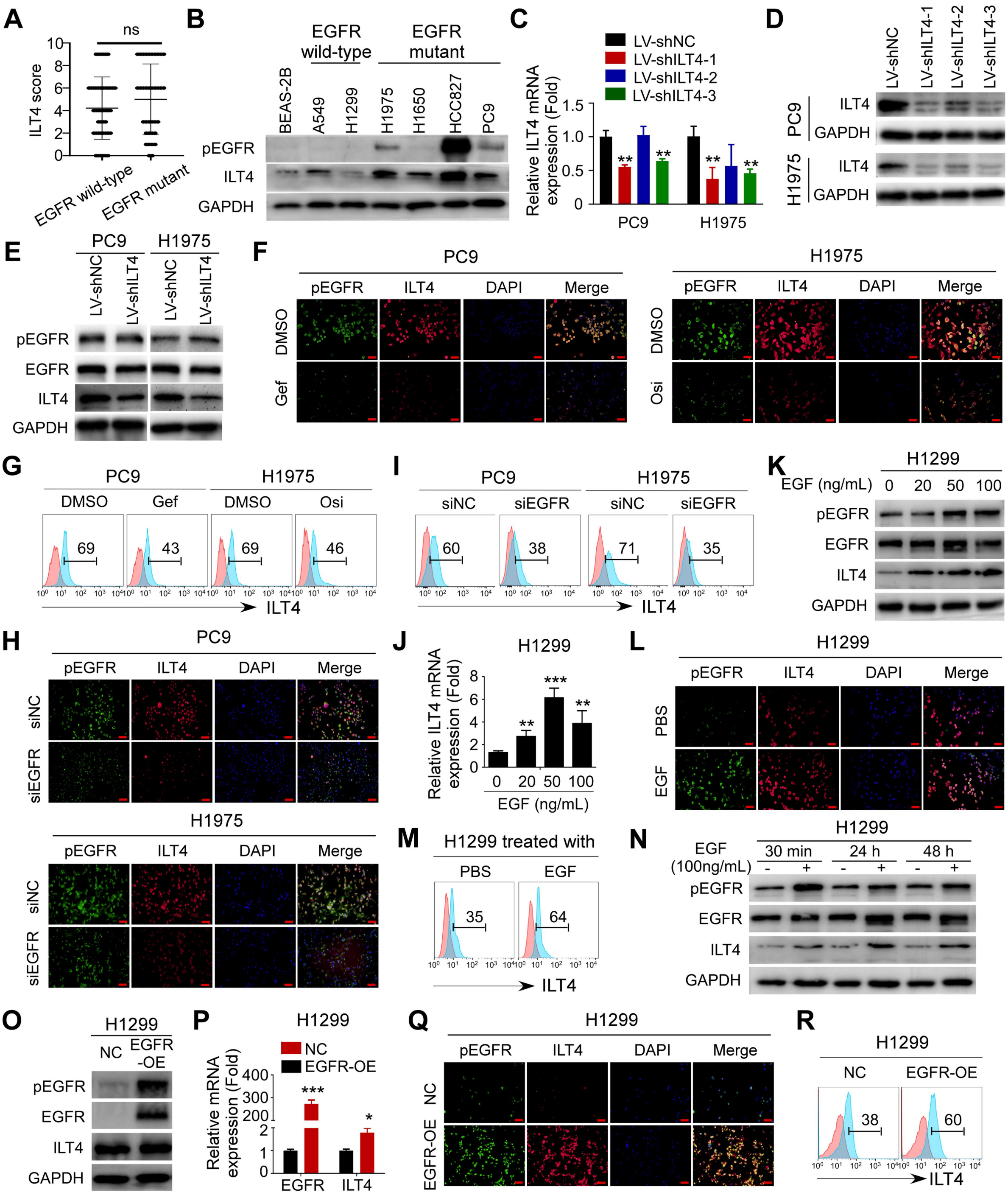

Figure S1. ILT4 expression in NSCLC cells was induced by EGFR activation

(A) The histogram shows result from 80 patients. There was no significant difference in ILT4 expression between EGFR mutant and wild-type groups by IHC analysis. **(B)** ILT4 expression in tumor cell lines was higher compared with that in bronchial epithelial cell line BEAS-2B. However, in EGFR mutant NSCLC cell lines, ILT4 and pEGFR levels were higher than EGFR wild-type cell lines. The expression of ILT4 and pEGFR was detected by Western blotting. **(C-D)** ILT4 expression was markedly downregulated by lentivirus carrying specific shRNA sequences. Both mRNA and protein expression of ILT4 in PC9 and H1975 cells was significantly downregulated upon transfection with ILT4-knockdown lentivirus as assayed by real-time PCR (C) and Western blotting (D). shILT4-1 and shILT4-3 showed the best efficiency. **(E)** The knockdown of ILT4 in PC9 and H1975 cells did not impact the expression of EGFR and pEGFR as evaluated by Western blotting. **(F-G)** Inhibition of EGFR activation by gefitinib or osimertinib significantly decreased ILT4 expression in PC9 or H1975 cells, respectively. PC9 and H1975 cells were treated with gefitinib (0.1 μM) or osimertinib (0.1 μM), respectively, for 24 h and immunofluorescence staining (F) and flow cytometry (G) were used to determined ILT4 and

pEGFR expression. **(H-I)** EGFR knockdown using specific siRNA suppressed ILT4 expression in PC9 and H1975 cells. PC9 and H1975 cells were transfected with EGFR- or control-siRNA for 72 h, and immunofluorescence staining (H) and flow cytometry (I) were used to analyze ILT4 and pEGFR levels. **(J-K)** EGF stimulation elevated ILT4 expression in H1299 cells in a concentration-dependent manner. H1299 cells were stimulated with 0, 20, 50, or 100 ng/mL EGF for 24 h before real-time PCR (J) and Western blotting (K). **(L-M)** ILT4 in H1299 cells was markedly upregulated by EGF stimulation. H1299 cells were treated with 100 ng/mL EGF for 24 h, and ILT4 and pEGFR expression was determined by immunofluorescence staining (L) and flow cytometry (M). **(N)** EGF stimulation elevated ILT4 expression in H1299 cells in a time-dependent manner. H1299 cells were stimulated with 100 ng/mL EGF for 30 min, 24 h, and 48 h before Western blotting. **(O-R)** EGFR overexpression in H1299 cells upregulated ILT4 expression at mRNA and protein levels. H1299 cells were transfected with EGFR overexpression plasmid for 48 h, and the mRNA expression of ILT4 was determined using real-time PCR (P) and protein expression by Western blotting (O), immunofluorescence staining (Q) and flow cytometry (R). *, $p < 0.05$; **, $p < 0.01$; ***, $p < 0.001$. Gef: gefitinib; NC: normal control; ns: no significance; OE: overexpression plasmid; Osi: osimertinib; si: siRNA.

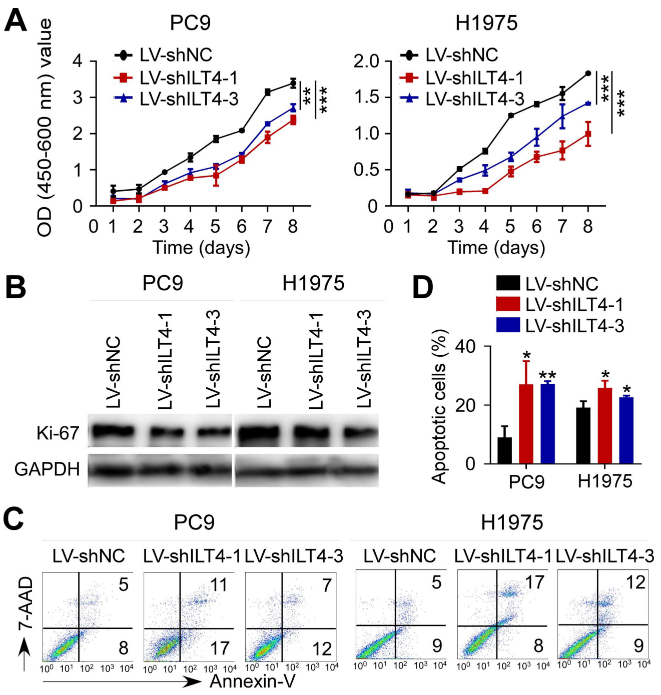

Figure S2. ILT4 knockdown inhibited the proliferation but induced apoptosis of EGFR-activated tumor cells

(A-B) ILT4 knockdown in PC9 and H1975 cells suppressed tumor cell proliferation. assessed by the CCK8 assay (A) and Ki-67 expression detected by Western blotting (B). **(C-D)** ILT4 knockdown in PC9 and H1975 cells promoted tumor cell apoptosis evaluated by flow cytometry after 48 h. (C) Flow cytometry and (D) Average results of 3 independent experiments. *, p < 0.05; **, p < 0.01; ***, p < 0.001. LV-shILT4: lentivirus carrying ILT4 shRNA; LV-shNC: lentivirus carrying control shRNA.

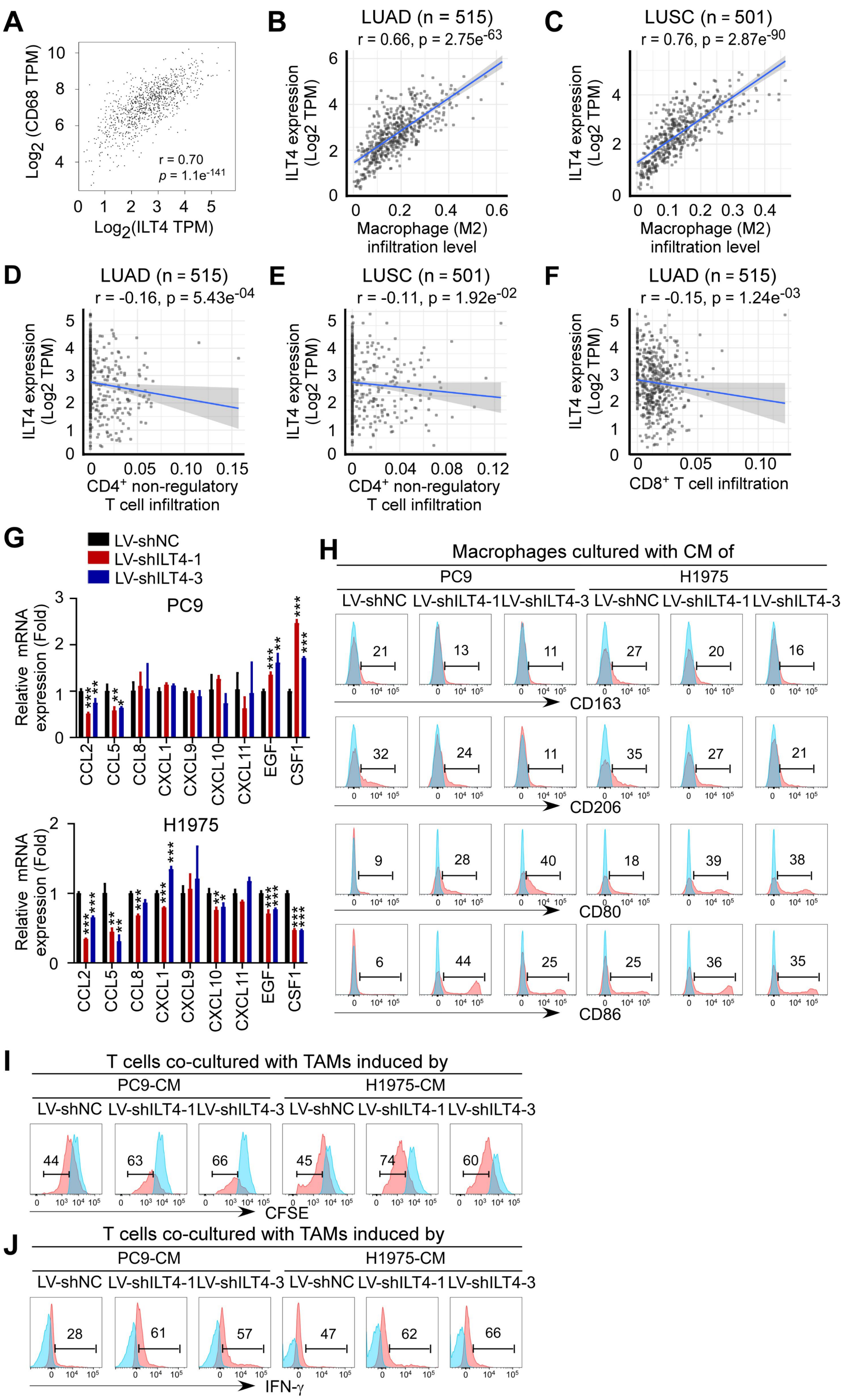

Figure S3. ILT4 expression in tumor cells was associated with increased M2-like TAM and decreased T cell infiltration in NSCLC

(A) ILT4 expression in NSCLC tissues was positively correlated with CD68 in the TCGA database. An online tool GEPIA was used to analyze the correlation between ILT4 and CD68 levels. **(B-F)** ILT4 expression was positively related to M2-like macrophage infiltration in lung adenocarcinoma (B) and squamous cell lung cancer (C), but negatively related to CD4+ and CD8+ T cell infiltration in lung adenocarcinoma (D-E) and squamous cell lung cancer (F). The immunedeconv R package [24] was utilized to make reliable immune infiltration estimations. **(G)** CCL2 and CCL5 expression was significantly decreased in ILT4 knockdown PC9 and H1975 cells. Several TAM-related cytokines expressed in tumor cells were

examined by real-time PCR. **(H)** ILT4 knockdown in tumor cells inhibited the M2-like polarization of co-cultured TAMs. (H) Images of flow cytometry in Figure 3L. **(I-J)** ILT4 knockdown promoted TAM-mediated T cell proliferation and IFN-γ generation. (I) Images of flow cytometry in Figure 3M. **(J)** Images of flow cytometry in Figure 3N.

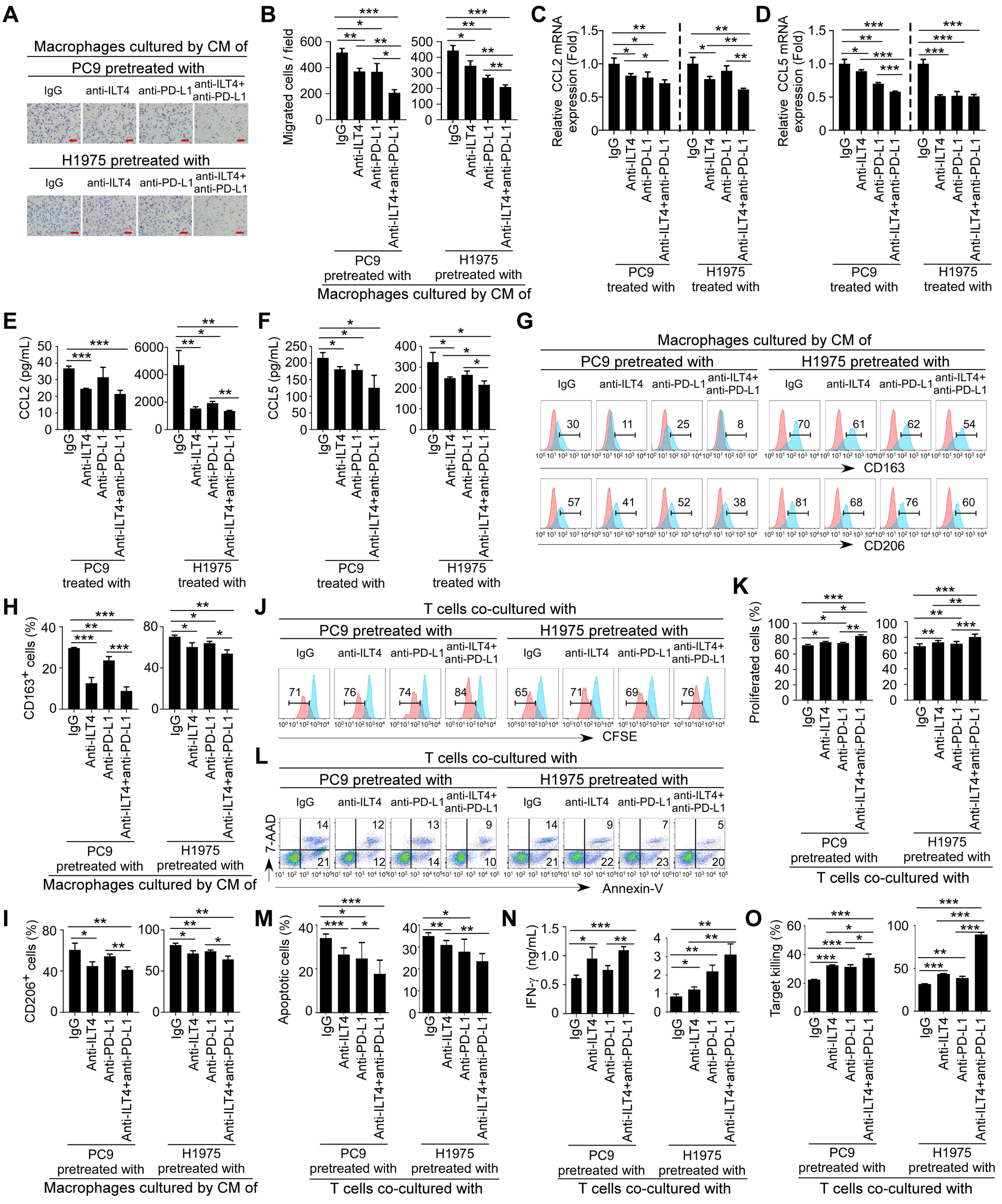

Figure S4. Combined ILT4 and PD-L1 blockade synergistically prevented M2-like TAM recruitment and T cell dysfunction in EGFR-activated tumor cells

(A-B) ILT4 or PD-L1 blockade in PC9 and H1975 cells restricted the migration ability of TAMs relative to the control IgG group while combination blockade revealed the most dramatic inhibition of TAM migration. The cell treatment and TAM induction were performed as in Figure 5A. TAM migration was detected by the Transwell migration assay. (A) Images of TAM migration, and (B) Average results from 3 independent experiments. Scale bar: 50 μm. **(C-F)** ILT4 or PD-L1 blockade in PC9 and H1975 cells decreased the expression and secretion of CCL2 and CCL5. (C-D) CCL2 and CCL5 mRNA expression detected by real-time PCR. (E-F) CCL2 and CCL5 secretion detected by ELISA. Tumor cells were treated and conditioned media were collected as described in (A). **(G-I)** ILT4 or PD-L1 blockade in PC9 and H1975 cells reversed tumor-induced the M2-like phenotype in TAMs, while combination blockade showed more significant reversion than either blockade alone. The same TAMs as in (A) were detected for M2-like marker expression using flow cytometry. (G) Representative results of CD163 and CD206 and (H-I) were average results from 3 independent repetitions. **(J-K)** ILT4 or PD-L1 blockade in PC9 and H1975 cells increased the proliferation ability of T cells, while combination therapy showed a synergistic increase in T cell proliferation. PC9 and H1975 cells were treated as mentioned in (A), and then co-cultured with anti-CD3-preactivated $CD3^+$ T cells for 4 days. Subsequently, CFSE proliferation assays were performed to determine the proliferation of T cells. (J) Images of flow cytometry and (K) Average results from 3 independent experiments. **(L-M)** ILT4 or PD-L1 blockade in PC9 and H1975 cells suppressed T cell apoptosis, while combined ILT4 and PD-L1 blockade showed the most significant suppression. T cells co-cultured with tumor cells as in (D) for 48 h were evaluated for apoptotic T cells by flow cytometry. (L) Representative results and (M) Average results from 3 independent experiments. **(N)** ILT4 or PD-L1 blockade in PC9 and H1975 promoted the secretion of IFN-γ in co-cultured T cells, while combined blockade released the most abundant IFN-γ. The T cells as in (L) were harvested and supernatants were used to evaluate IFN-γ levels by ELISA. **(O)** T cells co-cultured with anti-ILT4- or anti-PD-L1-pretreated PC9 and H1975 cells displayed stronger tumor eradication compared with those co-cultured with IgG-pretreated tumor cells, while T cells in the combination blockade group displayed the strongest cytolytic activity. Tumor cells were pretreated with different antibodies as mentioned in (A) before co-culturing with T cells. Cytolysis assays were employed to determine the killing ability of T cells. Anti: inhibitory antibody; CM: conditioned medium. *, $p < 0.05$; **, $p < 0.01$; ***, $p < 0.001$.

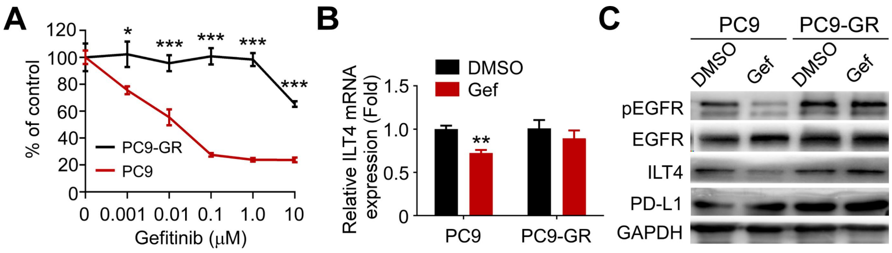

Figure S5. PC9-GR was resistant to gefitinib treatment

(A) Regular dose of gefitinib inhibited the proliferation of PC9 but not PC9-GR cells. The IC50 of PC9-GR was 15.16 μM, while that of PC9 was 0.018 μM. PC9 and PC9-GR cells were treated with different gefitinib concentrations for 24 h and the CCK8 assay was performed to determine tumor cell proliferation rate. **(B-C)** Gefitinib treatment downregulated ILT4 and pEGFR levels in PC9 rather than PC9-GR cells, which showed higher ILT4 expression than PC9. PC9 and PC9-GR cells were treated with 0.1 μM gefitinib for 24 h and ILT4 expression was detected by real-time PCR (B) and Western blotting (C). PD-L1, EGFR, and pEGFR expression was also evaluated by Western blotting. Gef: gefitinib. *, $p < 0.05$; **, $p < 0.01$; ***, $p < 0.001$.

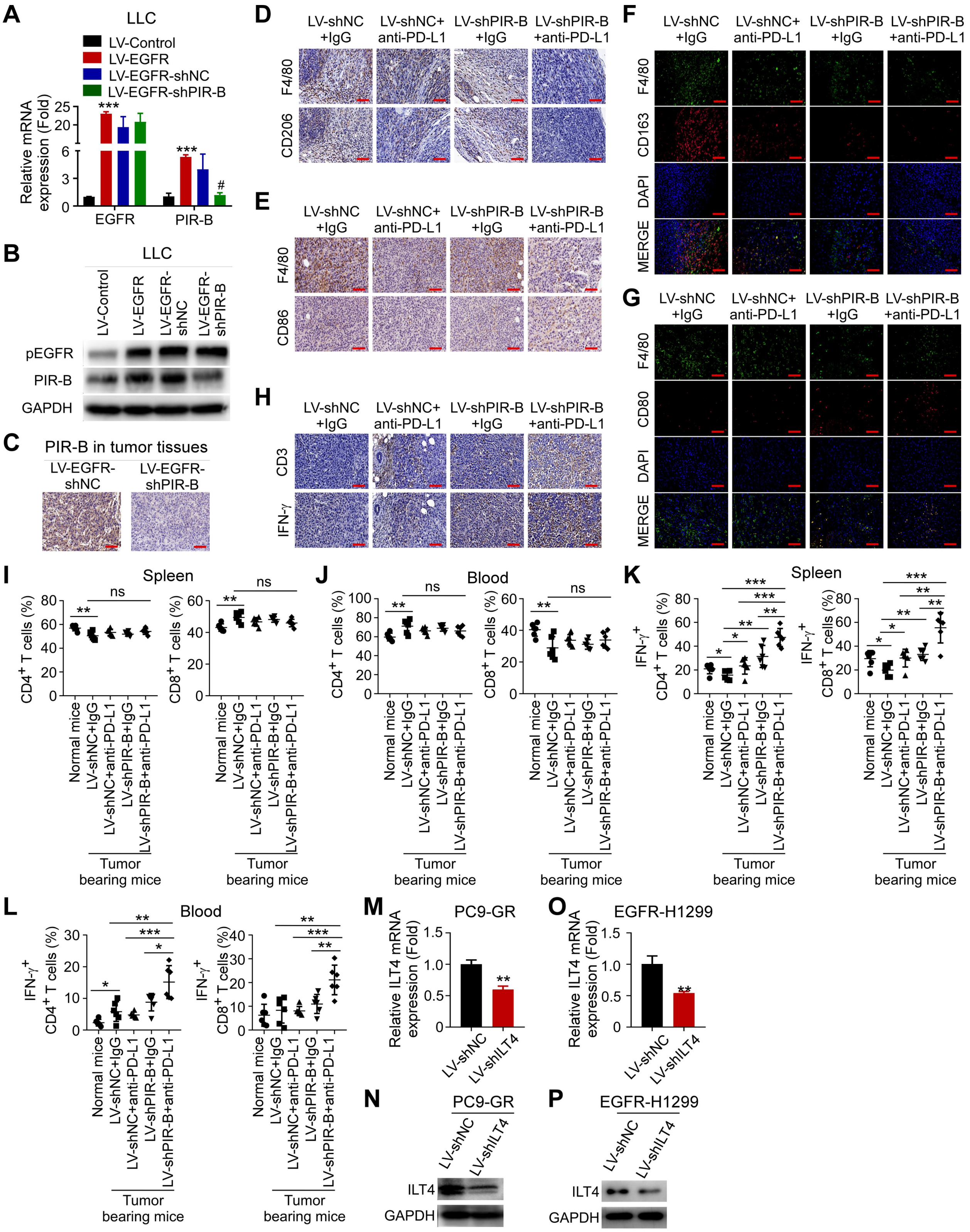

Figure S6. PIR-B and PD-L1 blockade synergistically prevented tumor growth and immune escape in vivo

(A-C) PIR-B expression was markedly downregulated by lentivirus carrying specific shRNA sequences in EGFR-activated LLC. The mRNA and protein expression of PIR-B in cell lines and mice tumor tissues were significantly decreased by real-time PCR (A), Western blotting (B), and IHC (C). **(D-G)** PIR-B or PD-L1 blockade markedly repressed TAM accumulation, decreased M2-like TAM, increased M1-like TAM frequencies in tumors, and the combined blockade group showed the strongest effect. (D-E) IHC and (F-G) Immunofluorescence staining. Tumor tissues were obtained from LLC-bearing C57BL/6 mice as described in Figure 6A. TAMs were defined as F4/80^{+} cells, M1-like TAMs were defined as

CD80^{+} and CD86^{+} cells, M2-like TAMs were defined as CD206^{+} and CD163^{+} cells. Scale bar: 20 μm. **(H)** PIR-B or PD-L1 blockade markedly induced CD3^{+} T cell accumulation and their IFN-γ expression in tumors, while combination therapy generated the most enriched CD3^{+} T cell infiltration and IFN-γ^{+} T cell frequency in tumor tissues. The tumor tissues were the same as in Figure 6A. Scale bar: 20 μm. **(I-J)** Tumor transplants decreased the CD4^{+} T subset and increased the CD8^{+} T subset in mouse spleens (I) and blood (J) detected by flow cytometry. However, the T cell subset composition in both organs of tumor-bearing mice was not regulated by inhibition of PIR-B or PD-L1 or both. The spleens and blood were the same as in Figure 6. **(K-L)** Spleens of tumor bearing mice showed lower IFN-γ^{+} CD4^{+} and IFN-γ^{+} CD8^{+} T cell frequency than normal tumor-free mice. However, PIR-B or PD-L1 blockade increased IFN-γ levels in both CD4^{+} and CD8^{+} T cells from mouse spleens (K) and blood (L), with the highest levels in the combination therapy group. The spleens and blood were the same as in Figure 6. **(M-P)** ILT4 expression was markedly downregulated by lentivirus carrying specific shRNA sequences in PC9-GR and EGFR-activated H1299 cells. The mRNA and protein expression of ILT4 was significantly decreased by real-time PCR (M, O) and Western blotting (N, P). *, $p < 0.05$; **, $p < 0.01$; ***, $p < 0.001$. Anti: inhibitory antibody; LV-shPIR-B: lentivirus carrying PIR-B shRNA; LV-shNC: lentivirus carrying control shRNA; ns: no significance.

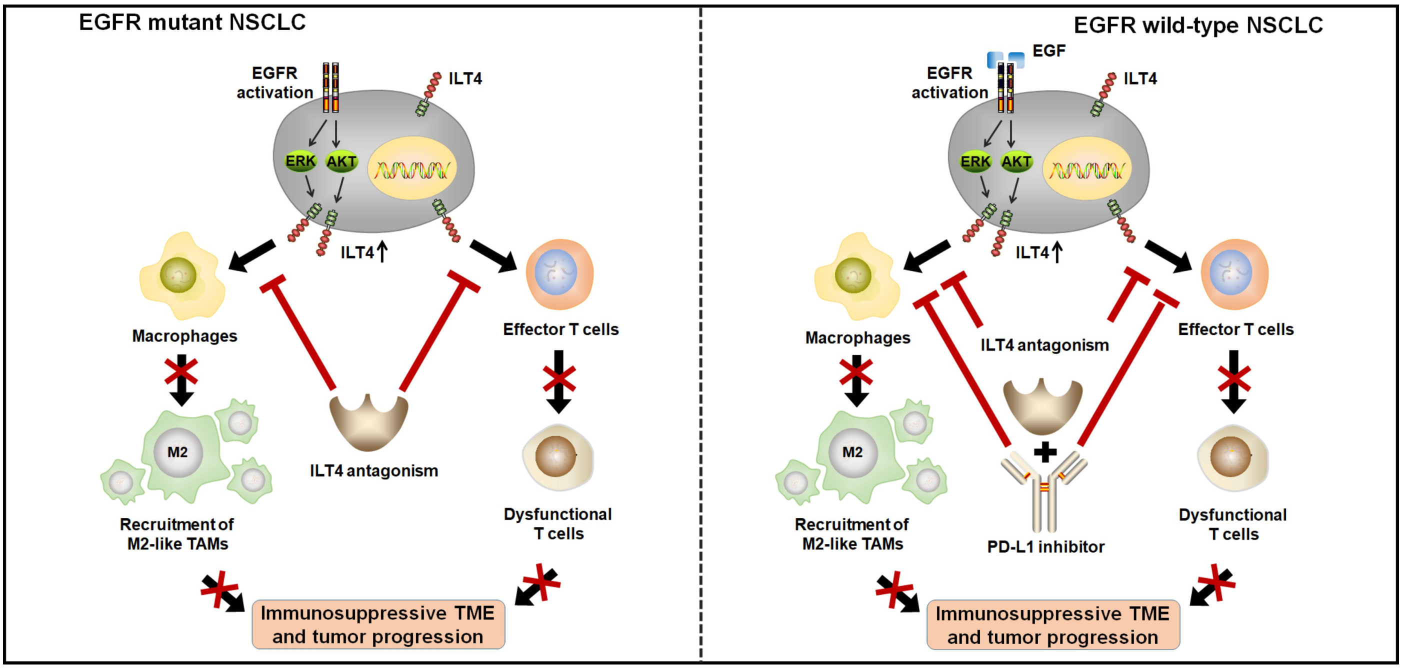

Figure S7. Schematic for ILT4-mediated immunosuppression and anti-tumor immunotherapy in NSCLC with EGFR activation.
